# New insights into bryophyte arabinogalactan‐proteins from a hornwort and a moss model organism

**DOI:** 10.1111/tpj.70312

**Published:** 2025-06-30

**Authors:** Kim‐Kristine Mueller, Lukas Pfeifer, Linus Wegner, Katrin Ehlers, Birgit Classen

**Affiliations:** ^1^ Pharmaceutical Institute, Department of Pharmaceutical Biology Christian‐Albrechts‐University of Kiel Gutenbergstraße 76 24118 Kiel Germany; ^2^ Institute of Botany Justus Liebig University Heinrich‐Buff‐Ring 38 35392 Giessen Germany

**Keywords:** Arabinogalactan‐protein, cell wall, *Anthoceros agrestis*, *Physcomitrium patens*, bryophyte, hornwort, moss, plant terrestrialization, electron microscopy

## Abstract

Two bryophyte models, the hornwort *Anthoceros agrestis* (*Anthoceros*) and the moss *Physcomitrium patens* (*Physcomitrium*), were analyzed for the presence of arabinogalactan‐proteins (AGPs), as the emergence of these signaling glycoproteins in evolution is still under debate. AGPs of both species had a galactan core structure similar to that of other bryophyte and fern AGPs, but different from angiosperm AGPs, as 1,6‐linked pyranosidic galactose was almost absent. In the *Physcomitrium* AGP, furanosidic arabinose (Ara*f*) linkages were mainly terminal (10%) or 5‐linked (13%), while in *Anthoceros*, terminal Ara*f* dominated (26%) and was accompanied by very low amounts of 1,3‐Ara*f* and pyranosidic terminal Ara. Unusual 3‐*O*‐methylated pyranosidic rhamnose, which has never been detected in cell walls of angiosperms, occurred in both bryophyte AGPs (5% in *Anthoceros*, 10% in *Physcomitrium* AGP). This was comparable to AGPs of other spore‐producing land plants. A bioinformatic search in the genomes of 14 bryophyte species revealed that most hornworts lack sequences encoding GPI‐anchored classical AGPs. Generally, hornworts contained fewer sequences for AGP protein backbones compared with the liverwort *Marchantia polymorpha* and the moss *P. patens*. All of them comprise sequences for chimeric AGPs, and among those, surprisingly xylogen‐like AGPs. Homologous sequences encoding glycosyltransferases and other enzymes involved in the synthesis and decoration of the AGP galactan framework were present in all bryophyte genomes. Immunocytochemistry of *Anthoceros* tissue detected AGPs at the plasma membrane/cell wall interface but also at the tonoplast, suggesting new functions of AGPs in bryophytes.

## Significance Statement

Extant bryophytes are key to infer evolution of the ost recent common ancestor of all land plants. As cell walls were important for adaptation to life on land, we analyzed AGPs from the hornwort *Anthoceros agrestis* and the moss *Physcomitrium patens* and detected terminal 3‐*O*‐methylrhamnose residues, which also occur in fern AGPs but not in angiosperms. Bioinformatic search for AGP protein backbones and glycosyltransferases in bryophyte genomes further strengthens understanding of AGP evolution during terrestrialization.

## INTRODUCTION

The split of the two major land plant clades, bryophytes, and tracheophytes, is dated more than 470 million years back (Yadav et al., 2023). Since then, the bryophytes with their remarkable biodiversity, formed by more than 20 000 extant species, adapted to almost every habitat (Degola et al., 2022; Morris et al., 2018; Wang et al., 2022). Recent phylogenomic evidence considers bryophytes as monophyletic (Bechteler et al., 2023; Wang et al., 2022; Zhang et al., 2020) with hornworts as sister clade to the so‐called setaphytes. The latter group consists of both bryophyte groups carrying a stalked spore capsule, namely, liverworts and mosses. As hornworts are the deepest‐diverging phylum of all extant land plants, they are important to reconstruct the evolution of land plants (Schafran et al., 2025). To investigate the molecular basis of various biological processes, many researchers supported the establishment of model organisms for each bryophyte clade. Milestones for this ambitious aim were the successive sequencing of genomes from all of the three bryophyte groups (Bowman et al., 2017; Li et al., 2020; Rensing et al., 2008; Schafran et al., 2025; Zhang et al., 2020).

Bryophytes show an extreme tolerance against abiotic stressors, like for example, changing light status, temperature and water availability (Bowles et al., 2021; de Vries & Archibald, 2018; Fürst‐Jansen et al., 2020; Yadav et al., 2023). This is reflected by the enormous diversity of the natural habitats, ranging from west tropical forests to the Arctic (Hodgetts et al., 2019; Wang et al., 2022). The extreme stress tolerance can also be seen as evolutionary heritage of the most recent common ancestor (MRCA) of all embryophyta. That MRCA had to cope with numerous environmental changes going hand‐in‐hand with plant terrestrialization and this flexibility formed the basis for the flourishing of all land plant taxa (de Vries & Archibald, 2018; Domozych & Bagdan, 2022; McCourt et al., 2023).

One of the many important adaptation steps was the modification of the cell wall (Harholt et al., 2016). This highly complex and dynamic system protects the cell from various stressors, both abiotic and biotic. Furthermore, it mechanically stabilizes the plant. The main components of the cell wall are polysaccharides—mainly cellulose, hemicelluloses, and pectic polysaccharides—as well as cell wall (glyco)proteins. All of these macromolecules were described to be generally present in bryophytes (Berry et al., 2016; Carafa et al., 2005; Geddes & Wilkie, 1971; Kremer et al., 2004; Pfeifer, Mueller, & Classen, 2022). Although a general similarity to tracheophyte walls is visible (for reviews, see Pfeifer, Mueller, & Classen, 2022; Ye & Zhong, 2022), in‐detail studies gave rise to some exciting new discoveries. Some examples are altered basic motifs in xyloglucans of mosses and liverworts (Peña et al., 2008) or arabinoglucans from *Physcomitrium patens*, which show structural features of mixed‐linked glucans (Roberts et al., 2018).

Cell wall (glyco)proteins are key players in plant cell wall biochemistry. The most dominant types are hydroxyproline‐rich glycoproteins (HRGPs). These can be further divided into arabinogalactan‐proteins (AGPs), extensins (EXT), and proline‐rich proteins (PRPs) with differing degrees of glycosylation. While AGPs show huge glycan parts of around 90% of the molecule, only 50% of EXTs and even lower portions of PRPs are built up of carbohydrates (Johnson et al., 2018). Both moieties (protein and carbohydrates) are covalently connected *via* the 4‐*O*hydroxylated derivative of proline (=hydroxyproline, Hyp). Glycosylation in the polysaccharide part shows the characteristics of type II arabinogalactans, formed by (1 → 3)‐linked pyranosidic β‐d‐galactose (Gal*p*) units which are connected to (1 → 6)‐β‐d‐galactan side chains at position O‐6. This galactan backbone is further decorated with furanosidic α‐l‐arabinose (Ara*f*) and other monosaccharides in minor amounts, such as l‐rhamnose (Rha), l‐fucose (Fuc) or d‐glucuronic acid (GlcA; Kitazawa et al., 2013; Strasser et al., 2021). The distribution of AGPs among land plants is widespread and also bryophytes were investigated very early in the 1970s by Clarke et al. (1978). The authors used the interaction between the AGP glycan part and β‐glucosyl Yariv reagent for precipitation (βGlcY; Yariv et al., 1962). AGPs from all major land plant lineages, that is, ferns, gymnosperms, as well as monocots and dicots (Bartels & Classen, 2017; Baumann et al., 2021; Classen et al., 2004; Goellner et al., 2011; Mueller et al., 2023) were analyzed over the last decades. Furthermore, few studies focused on bryophytes, but characterized only AGPs of mosses and liverworts (Bartels et al., 2017; Fu et al., 2007; Happ & Classen, 2019; Lee et al., 2005) and not of hornworts. Beside a general similarity of spore plant AGPs to angiosperm AGPs, significant structural peculiarities, namely, high amounts of methylated rhamnose (3‐*O*‐MeRha; acofriose; Bartels & Classen, 2017; Bartels et al., 2017; Fu et al., 2007; Happ & Classen, 2019; Mueller et al., 2023), were found.

Seed plant AGPs are involved in many developmental processes, including xylem differentiation, cell proliferation, growth, salt and drought tolerance, as well as interaction with plant microbes (Ma et al., 2018; Mareri et al., 2018; Seifert & Roberts, 2007). Bryophyte AGPs were described to contribute to water balance in mosses (Cui et al., 2012; Kobayashi et al., 2011; Ligrone et al., 2002) as well as to apical cell extension in protonemata of the moss *P. patens* (Lee et al., 2005).

In this study, we hypothesize that AGPs in bryophytes are important for the successful adaptation to life on land. Bryophyte AGPs of the model moss *P. patens* and the model hornwort *A. agrestis* were purified to further understand the nature of AGP glycans in bryophytes. No hornwort AGP has been isolated and characterized up to now. Our investigations are complemented by immunocytochemical detection of AGPs in hornwort tissue. Bioinformatic search for AGP protein backbones and glycosyltransferases in genomes of all bryophyte lineages (Bowman et al., 2017; Rensing et al., 2008; Schafran et al., 2025) further strengthens understanding of AGP evolution during terrestrialization.

## RESULTS

### Gel diffusion assay

A gel diffusion assay was used to search for AGPs in the high molecular weight, water‐soluble fractions (AE) of the bryophytes. AGPs contain 1,3‐galactan chains that precipitate with red‐colored Yariv phenyl glycosides, such as β‐d‐glucosyl Yariv reagent (βGlcY; Kitazawa et al., 2013; Paulsen et al., 2014). This precipitation can be perceived as a red line in a gel diffusion assay (Clarke et al., 1979) and was positive for *Anthoceros* and *Physcomitrium* AEs, comparable to AE from *Marchantia polymorpha* (Figure S1), which has been investigated in a previous study (Happ & Classen, 2019).

### Isolation and yield of AGPs

The extraction of the high molecular weight, water‐soluble fractions (AE) yielded 12.0 ± 3.1% for *Anthoceros* plants (*n* = 2), 10.9% for *Anthoceros* cell suspension culture, and 2.1% of the dry plant material for *Physcomitrium* (*n* = 1). The fractions comprised different monosaccharides, with Glc, Gal, Xyl, and Ara dominating in these fractions (Tables S1 and S2). In both AE samples from *Anthoceros* (plant and cell culture), Gal and Ara accounted for over 50% of the neutral monosaccharides, with higher amounts of Ara in the cell culture. In *Physcomitrium*, Glc was dominant, followed by Gal and Ara. It must be taken into account that at least part of the Glc probably originates from starch due to the lack of amylase treatment (Figure S2).

Based on the high amount of Ara and Gal in the AE fractions (Table S1) as well as the positive gel diffusion assays (Figure S1), the AE fractions were treated with βGlcY‐reagent to gain AGP fractions. The yields of the *Anthoceros* AGP fractions were very high with 0.31% ± 0.08% (plants, *n* = 2) and 0.57% (cell culture, *n* = 1) of the dry plant material compared to that of *Physcomitrium* (0.09%, *n* = 1).

### Carbohydrate moiety of bryophyte AGPs

The neutral monosaccharides Ara and Gal were clearly dominant in *Anthoceros* AGP fractions (Table 1), accounting together for 85.7% (plant) and 90.1% (cell culture). The content of Ara was remarkably high in AGP from *Anthoceros* cell culture, leading to a ratio of 1:0.8 (Ara:Gal) which is unusual for AGPs. In contrast, these ratios in AGPs from *Anthoceros* plants and *Physcomitrium* were quite similar and typical for AGPs (1:1.7 and 1:1.6). High amounts of Glc were additionally present in *Physcomitrium* AGP. Rha was part of all AGPs with a higher amount in *Physcomitrium* and mainly present as 3‐*O*‐methyl derivative (3‐*O*‐MeRha).

**Table 1:** Neutral monosaccharide composition of AGPs from *Anthoceros agrestis* plants and cell cultures and *Physcomitrium patens* in % (mol mol^−1^)

Neutral monosaccharide	*Anthoceros* (plant)		*Anthoceros* (cell culture)	*Physcomitrium*	
	AGP (*n* = 1)	AGP_UR_ (*n* = 3)	AGP (*n* = 3)	AGP (*n* = 1)	AGP_UR_ (*n* = 2)
3‐*O*‐Me‐Rha	5.6	4.3 ± 0.1	5.1 ± 0.1	10.0	10.6 ± 0.4
Rha	tr	tr	tr	3.4	2.8 ± 0.1
Fuc	tr	tr	tr	2.0	1.5 ± 0.1
Ara	31.8	35.6 ± 0.7	49.3 ± 0.6	21.8	21.1 ± 0.0
Xyl	4.0	3.3 ± 0.4	tr	2.8	2.7 ± 0.3
Man	1.6	1.9 ± 0.3	3.4 ± 0.1	3.0	2.4 ± 0.0
Gal	53.9	50.3 ± 0.8	40.8 ± 0.5	35.2	35.6 ± 0.0
Glc	3.1	4.6 ± 1.0	1.4 ± 0.1	21.8	23.3 ± 0.0
Ara:Gal	1:1.7	1:1.7	1:0.8	1:1.6	1:1.7

Abbreviation: tr, trace value <1%.

The uronic acid content of the AGPs determined by colorimetric quantification was comparable between 5 and 7% (Table S2). Furthermore, AGPs were subjected to uronic acid reduction with sodium borodeuteride (AGP_UR_). In the mass spectrum analysis, deuterated fragments were detected only in the peak of Glc (see below, Table 2). This, together with the slight increase of Glc in the neutral monosaccharide analysis after uronic acid reduction (Table 1, AGP_UR_) identified GlcA as part of bryophyte AGPs.

**Table 2:** Linkage‐type analysis of AGP and AGP_UR_ samples from *Anthoceros agrestis* (plant) and *Physcomitrium patens* in % (mol mol^−1^, *n* = 1)

Monosaccharide	Linkage type	*Anthoceros*		*Physcomitrium*	
		AGP	AGP_UR_	AGP	AGP_UR_
Gal*p*	1,3,6‐	28	24	22	18
	1,3,4‐	7	7	‐	‐
	1,6‐	Tr	1	1	1
	1,3‐	20	18	11	11
	t‐	1	1	2	2
Glc*p*	1,4‐	‐	2^a^	23	20
	t‐	1	2^a^	2	4^a^
Man*p*	1,6‐	Tr	tr	tr	2
Hex*p*	1,3,4,6‐	7	7	‐	‐
Rha*p*	t‐	8	7	17	18
Fuc*p*	t‐	‐	‐	1	1
Ara*f*	1,5‐	Tr	tr	11	13
	1,3‐	2	2	tr	tr
	t‐	24	26	10	10
Ara*p*	t‐	2	2	tr	tr
Xyl*p*	t‐	Tr	1	tr	tr

tr, trace value <1%; UR, uronic acid‐reduced.

^a^ Including deuterated fragments.

### Structure elucidation of the arabinogalactan (AG) moieties of bryophyte AGPs

**Figure 1:**
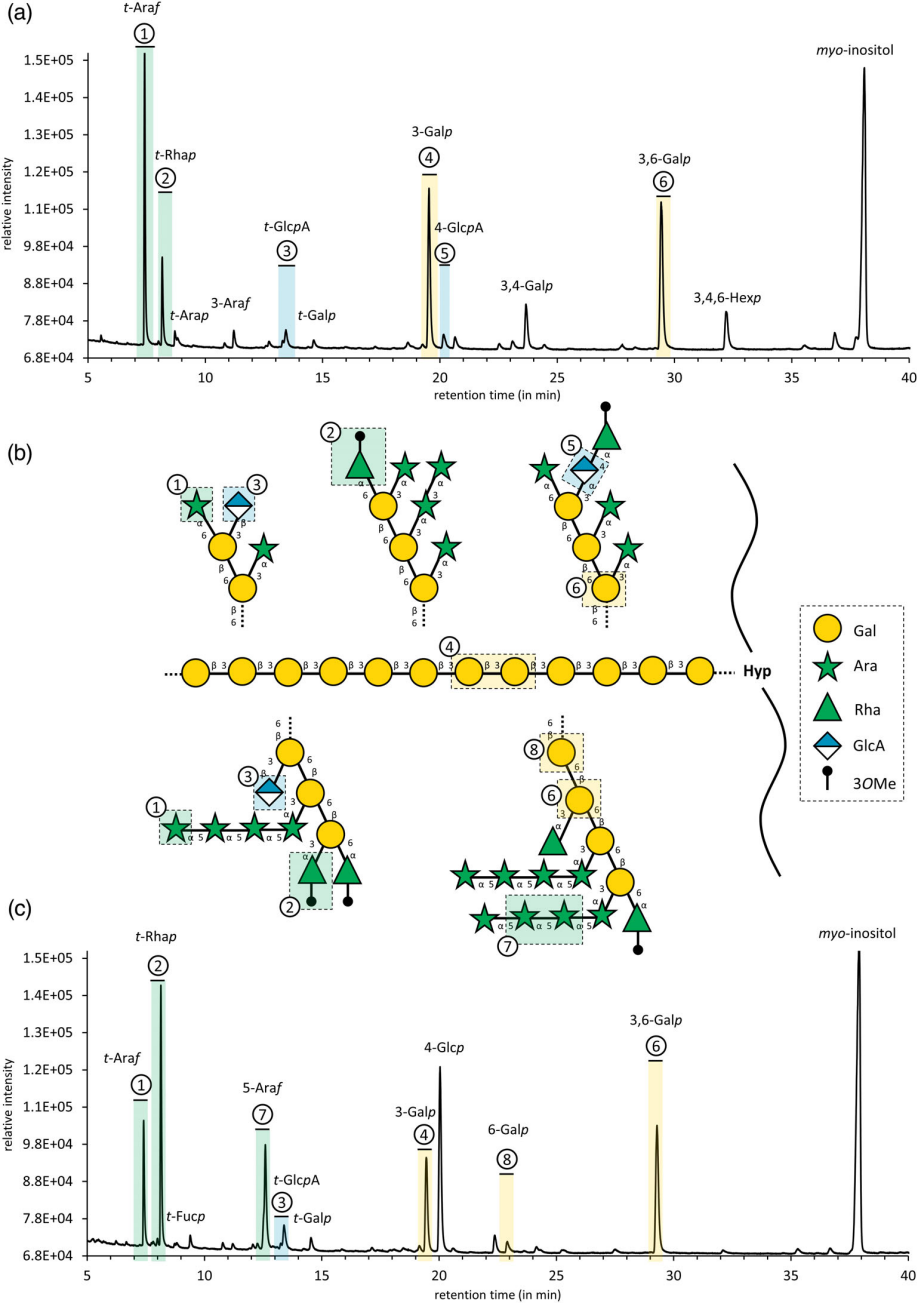
Results of the linkage‐type analysis of *Anthoceros agrestis* and *Physcomitrium patens* AGPs. The highlighted structural features are numbered with the same numbers in all three panels of the diagram. (a) Gas chromatogram of methylated and *per*‐acetylated monosaccharides derived from *Anthoceros* AGP_UR_. (b) Structural proposal of fragments inferred from linkage analysis. The upper half of this panel represents *Anthoceros* fragments while the lower half represents *Physcomitrium* fragments. The central 1,3‐linked Gal*p* backbone is shared. (c) Gas chromatogram of methylated and *per*‐acetylated monosaccharides derived from *Physcomitrium* AGP_UR_.

For structural characterization, the AGPs and the uronic acid‐reduced AGPs (AGP_UR_) were subjected to linkage‐type analysis (Table 2). Figure 1 shows the GCchromatograms of both samples after *per*‐methylation and acetylation together with structural proposals for *Anthoceros* and *Physcomitrium* AGPs. In all samples, Gal*p* was the main monosaccharide and was present mainly in 1,3,6 linkage (18–28%) and 1,3 linkage (11–20%). In *Anthoceros* AGP, 1,3,4‐linked Gal was present additionally. The linkage types of Ara also differed in the two investigated bryophytes. In *Anthoceros* AGP, terminal Ara*f* was strongly dominant (24%/26%), whereas in *Physcomitrium* AGP, more 1,5‐Ara*f* was present and accompanied by almost similar amounts of terminal Ara*f*.

Further terminal monosaccharides of both AGPs were t‐Rha*p* with up to 18% in *Physcomitrium*, terminal Gal*p*, and terminal Glc*p*A. In the AGP samples, uronic acids are not detectable due to the method used. After reduction of uronic acids with borodeuteride (AGP_UR_), deuterated fragments were detected in the peak of terminal Glc, and the amount of this peak slightly increased, indicating the presence of terminal GlcA in both native AGPs (Table 2). Further deuterated fragments were found in 1,4‐linked Glc present in *Anthoceros* AGP in small amounts, possibly originating from 1,4‐GlcA. Additionally, a 1,3,4,6‐Hex*p* peak was identified in *Anthoceros* AGP. Since it cannot be clearly determined whether this is an artificial peak due to undermethylation, it was not included in the structural proposal (Figure 1). In the *Physcomitrium* AGP sample, high amounts of 1,4‐linked Glc (20%) were detected. As this might originate from accompanying starch, it was also not included in the structural proposal (Figure 1). Fuc, Man, and Xyl were not found in significant amounts in both bryophyte AGPs.

### Characterization of AG epitopes with antibodies

**Figure 2:**
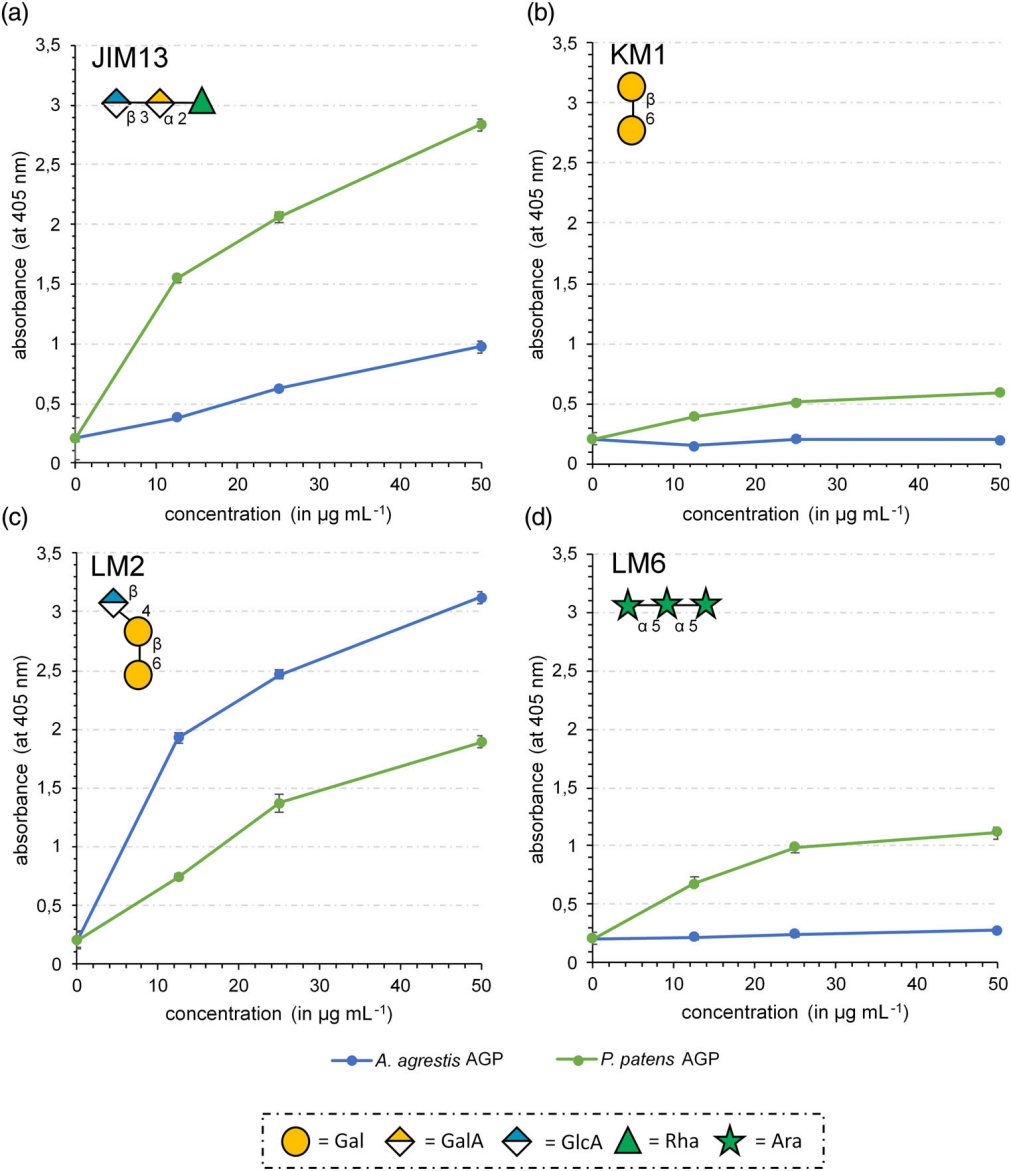
Reactivity of AGPs of *Anthoceros agrestis* (blue) and *Physcomitrium patens* (green) with antibodies against epitopes of AGP glycans tested by ELISA. (a) JIM13; (b) KM1; (c) LM2; (d) LM6. For epitopes of the antibodies, see Table S3.

To obtain more information about the structure of the AG moieties of the two bryophyte AGPs, they were tested by ELISA for their binding affinity to different antibodies directed against AGP glycan epitopes (JIM13, KM1, LM2 and LM6; Figure 2; Table S3). The antibody JIM13 (Figure 2a; Pfeifer, Utermöhlen, et al., 2022; Yates et al., 1996) is generally considered to bind to AGPs, and the trisaccharide structure β‐d‐Glc*p*A‐(1 → 3)‐α‐d‐Gal*p*A‐(1 → 2)‐α‐l‐Rha has been suggested as an epitope. The AGPs of *Physcomitrium* showed a strong and that of *Anthoceros* a moderate affinity to JIM13. KM1, directed against (1 → 6)‐β‐d‐Gal*p* units in AGs type II (Figure 2b; Classen et al., 2004; Ruprecht et al., 2017) did not bind to the *Anthoceros* AGPs and had very weak reactivity with the *Physcomitrium* AGPs. The antibody LM2 (Smallwood et al., 1996, Figure 2c), identifying (1 → 6)‐β‐d‐Gal*p* units with terminal β‐d‐Glc*p*A in AGPs (Ruprecht et al., 2017), reacted strongly with both bryophyte AGPs, but even more with *Anthoceros* AGP. Only the AGP of *Physcomitrium* showed a moderate binding to the antibody LM6 (Figure 2d) which detects oligomers with (1 → 5)‐α‐l‐Ara*f* linkages in arabinans or AGPs (Verhertbruggen et al., 2009). Thus, the ELISA experiments underlined the structural differences between the hornwort and the moss AGPs (Figure 1).

### Enzymatic toolkit for AGP glycosylation in bryophytes

**Figure 3:**
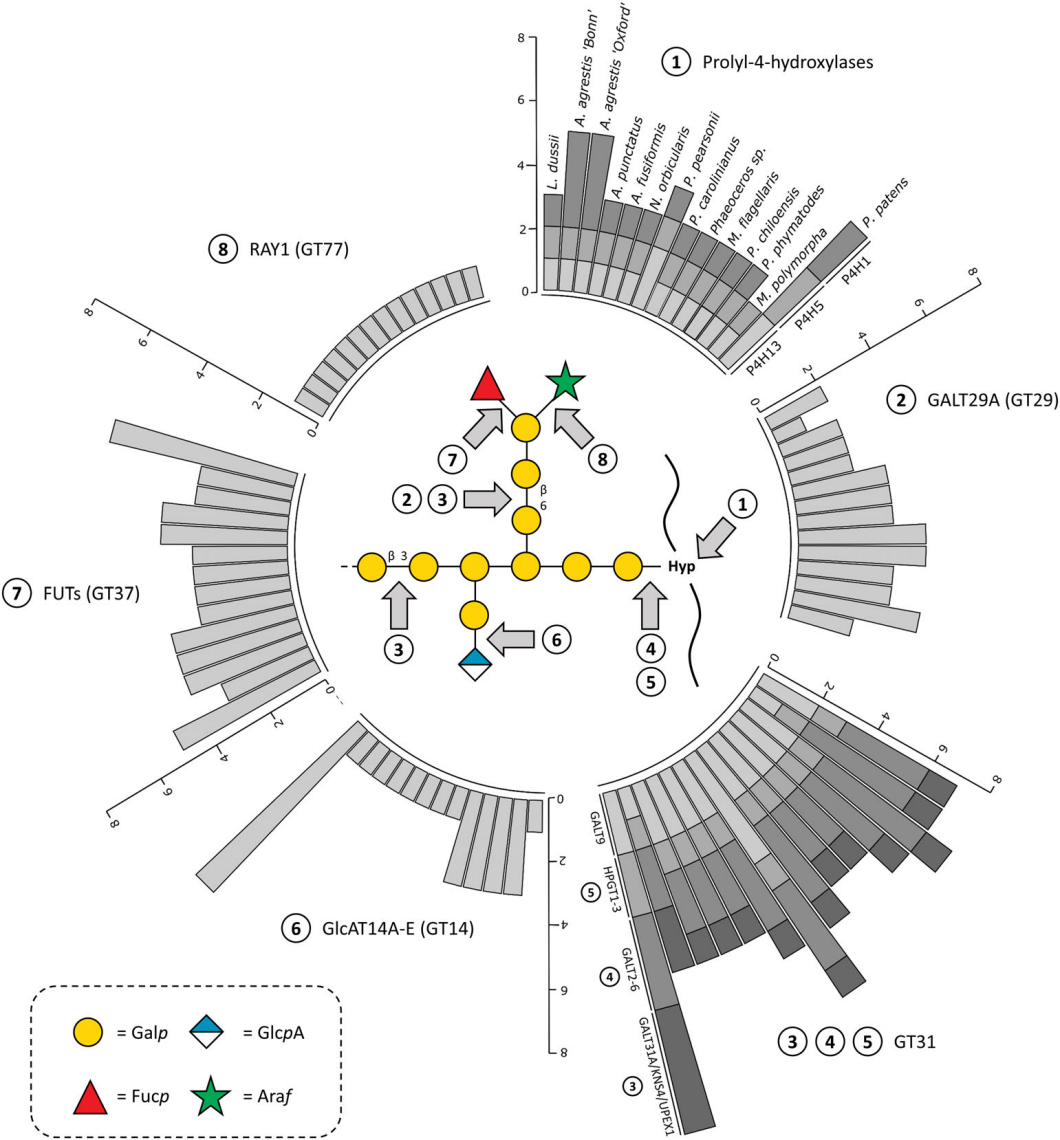
Circular stacked bar plot showing homolog numbers of characterized enzymes involved in AGP biosynthesis. A hypothetical model of AGP structure based on published enzyme specificities is shown in the middle. Gray arrows represent linkages resulting from the respective enzyme activities. Hornworts: *Leicosporoceros dussii*, *Anthoceros agrestis* ‘Bonn’, *Anthoceros agrestis* ‘Oxford’, *Anthoceros punctatus*, *Anthoceros fusiformis*, *Notothylas orbicularis*, *Paraphymatoceros pearsonii*, *Phaeoceros carolinianus*, *Phaeoceros* sp., *Megaceros flagellaris*, *Phaeomegaceros chiloensis*, *Phymatoceros phymatodes*; liverwort: *Marchantia polymorpha*; moss: *Physcomitrium patens*. For phylogenetic trees of all enzyme families, see Data S1–S12.

Twelve hornwort genomes (Schafran et al., 2025) as well as the genomes of *M. polymorpha* (Bowman et al., 2017) and *P. patens* (Lang et al., 2018; Rensing et al., 2008) were searched for enzyme homologs involved in AGP glycosylation (Figure 3; Table S4; Data S1–S12). As described above, glycosylation of AGPs is very complex, including a galactan framework attached to the protein moiety *via* Hyp and further decoration of the galactan with mainly Ara and different other monosaccharides, such as GlcA, Rha, Fuc, or Xyl (for reviews, see Showalter & Basu, 2016; Silva et al., 2020; Strasser et al., 2021). Prolyl‐4‐hydroxylases (P4Hs) are responsible for hydroxylation of proline. Phylogenetic analysis revealed the presence of three to five putative AGP‐related P4H homologs in hornwort genomes; *Marchantia* contained only two, whereas *Physcomitrium* had six (Data S1). Furthermore, we searched for glycosyltransferases (GTs, Data S3–S7) and glycosylhydrolases (GHs, Data S8–S12) involved in AGP biosynthesis and remodeling, which are classified according to carbohydrate‐active enzyme nomenclature (CAZy; http://www.cazy.org). Several members of the GT31 family are described to be responsible for AGP galactosylation. *Arabidopsis* GALT 2–6 (Basu, Tian, et al., 2015; Basu, Wang, et al., 2015) as well as HPGT1‐3 (Ogawa‐Ohnishi & Matsubayashi, 2015) are responsible for the first step of AGP glycosylation: the transfer of the initial Gal onto Hyp. Genomes of all hornworts, *Marchantia*, and *Physcomitrium* had corresponding sequences in comparable numbers (three to five sequences). GALT31A, UPEX1/KNS4, GALT9 (family GT31), and GALT 29A (family GT29) are involved in the synthesis of the 1,3‐, 1,6‐linked galactan framework in *Arabidopsis*, and all bryophyte genomes contained homologs of these sequences in comparable numbers (one to four sequences, with a high number of 4 GALT31A, UPEX1/KNS4 homologs in *Physcomitrium*).

Members of GT14 family of glucuronosyltransferases are acting on AGPs, especially GlcAT14A‐E, which adds terminal Glc*p*A to the AG moieties. Homologs were detected in all bryophyte genomes with lower numbers in hornworts and the liverwort (one to three homologs) compared with *Physcomitrium* (7 homologs).

Arabinosyl‐ and rhamnosyltransferases responsible for the decoration of the galactan backbone of AGPs are mainly unknown up to now (Silva et al., 2020). Only one homolog of the β‐arabinofuranosyltransferase RAY1 (Gille et al., 2013) is present in all investigated hornwort and setaphyte genomes. No enzyme homologs of AGM1 + 2 occur in bryophyte genomes (Data S2). This corresponds to our biochemical data, as these enzymes transfer methylethers at C4 of GlcA in *Arabidopsis* AGPs (Temple et al., 2019) and we did not find any methylated GlcA in bryophyte AGPs. Homologs of GT37 occur in all bryophytes and comprise fucosyltransferases. As bryophyte AGPs contain no or only traces of Fuc (see Tables 1 and 2), members of the GT37 family possibly mainly act on other polysaccharides in bryophytes.

GHs known to be involved in AGP remodeling are RsAraf (GH3), AGAL2‐3 (GH27), AtBGAL8 (GH35), AtGUS2 (GH79), and members of GH43. Homologous protein sequences coding for these enzymes were present in all bryophytes.

### Protein moiety of bryophyte AGPs

Since AGPs consist of a protein moiety covalently linked *via* Hyp to the carbohydrate part, the protein and the Hyp content of the bryophyte AGPs were determined. From the nitrogen content measured by elemental analysis (2.6% for *Anthoceros* and 2.7% for *Physcomitrium* AGP), the amount of protein in the AGPs was calculated using the Kjeldahl factor (× 6.25) and determined to be 16.3 and 16.9% of the dry weight of the AGPs, respectively. The Hyp amount of the *Anthoceros* AGP was slightly higher (0.31%) compared to that of *Physcomitrium* (0.25%). This results in a Hyp proportion of 1.9% of the protein in *Anthoceros* AGP and 1.5% for that of *Physcomitrium*.

### AGP protein sequences present in bryophyte genomes

**Figure 4:**
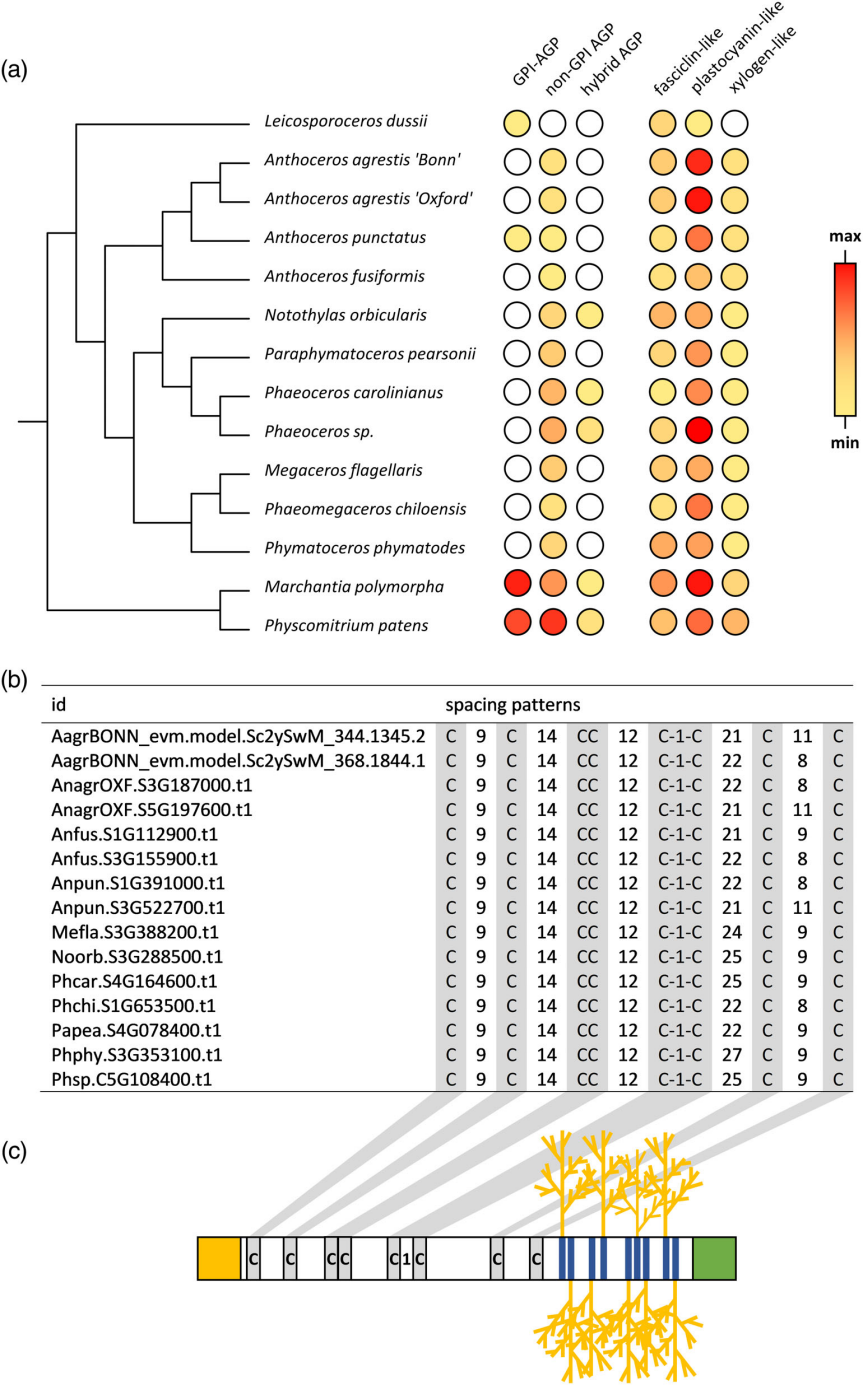
Identified AGP protein backbone sequences in 14 bryophyte genomes (12 hornworts, 1 liverwort, and 1 moss). (a) Heatmap representation of numbers for classical AGPs (GPI‐anchored, non‐GPI‐anchored), hybrid AGPs, as well as selected chimeric AGPs (fasciclin‐like, plastocyanin‐like, and xylogen‐like). (b) Detailed structural analysis of spacing patterns between the eight conserved cysteine residues in the nsLTP2‐domain (pfam14368) being characteristic for xylogen‐like chimeric AGPs. (c) Illustration of xylogen‐like AGPs with the conserved cysteine residues shaded in gray, AG‐glycomotifs shaded in blue. The *N*‐terminal signal peptide and a GPI‐anchor sequence are represented as yellow and green squares, respectively.

Bryophyte genomes were also used to search for genes coding for AGP protein backbones. Sequences for classical AGPs with and without GPI‐anchor as well as hybrid AGP sequences with additional motifs from other groups of HRGPs were identified (Figure 4a; Table S5) using the established motif and amino acid bias ‘MAAB’ classification system (Johnson, Cassin, Lonsdale, Bacic, et al., 2017; Johnson, Cassin, Lonsdale, Ka‐Shu Wong, et al., 2017). It is striking that 10 of the hornwort genomes completely lack sequences for GPI‐anchored classical AGPs; only the two genomes of *Leicosporoceros dusii* and *Anthoceros punctatus* contain one of these sequences. This is in strong contrast to the liverwort *M. polymorpha* (20 sequences) and the moss *P. patens* (16 sequences). All hornworts with the exception of *L. dusii* possess genome sequences for AGPs without GPI‐anchor (1–7 sequences, in mean 2.9 sequences). These GPI‐free AGPs are also more abundant in the setaphytes (*Marchantia* with nine sequences; *Physcomitrium* with 18 sequences). Only few hybrid AGPs with an additional extensin or PRP motif were present in the investigated bryophyte genomes. The hornworts *Notothylas orbicularis*, *Phaeoceros* sp. and *Phaeoceros carolinianus* as well as *Marchantia*, and *Physcomitrium* had one or two sequences, whereas all other hornwort genomes lacked hybrid AGP sequences.

In chimeric AGPs (Figure 4a; Table S6), AGP motifs are accompanied by a signal peptide and one or more known functional protein domains (as classified in the pfam database, http://pfam.xfam.org/). Typical chimeric AGPs known from angiosperms were detected in the bryophyte genomes, namely, plastocyanin‐like AGPs (containing the Cu bind‐like domain, pfam02298), fasciclin‐like AGPs (containing the FAS1 domain, pfam02469) and xylogen‐like AGPs (containing the LTP‐2 domain, pfam14368; Ma et al., 2017). Most abundant in all bryophytes were the plastocyanin‐like AGPs with in mean 11.2 sequences in hornworts, 21 sequences in *Marchantia* and 13 sequences in *Physcomitrium* (Figure 4a; Table S6). In comparison to the hornworts (range 1–7, in mean 3.4 sequences), the fasciclin‐like AGPs were slightly more abundant in *Marchantia* (9 sequences) and *Physcomitrium* (5 sequences). Within the bryophytes, xylogen‐like AGP sequences dominated in the setaphytes (*Marchantia* with three sequences, *Physcomitrium* with six sequences); but some also occurred in the hornworts (one or two sequences, no sequence in *Leicosporoceros*). Xylogen‐like AGP sequences contain lipid‐transfer‐like domains (Figure 4b,c), which only occur in land plants and are very stable due to four disulfide bridges formed by eight conserved cystein residues (Edstam & Edqvist, 2014). Detailed analyses of this nonspecific lipid transfer protein (nsLTP) domain was performed by looking at the spacing patterns between the eight conserved cysteines (Figure 4b; Table S7) together with a check for putatively present GPI‐anchors. These analyses revealed presence of only the G and G/D types (Figure 4b,c; for detailed classification of nsLTPs, see Liu et al., 2015). Further comparison with other genomic data (2 angiosperms, 2 gymnosperms, 3 ferns, 2 lycophytes, *Physcomitrium* and *Marchantia*) confirmed sole occurrence of these domain types (Table S8) in xylogen‐like AGPs. G‐type LTPs were initially classified by the presence of GPI‐anchors as described in Edstam et al. (2011). It has to be noted that the sequences labeled with “G/D” could possibly be G‐type LTPs lacking these anchor structures.

### Immunocytochemical detection of *Anthoceros* AGPs by TEM

**Figure 5:**
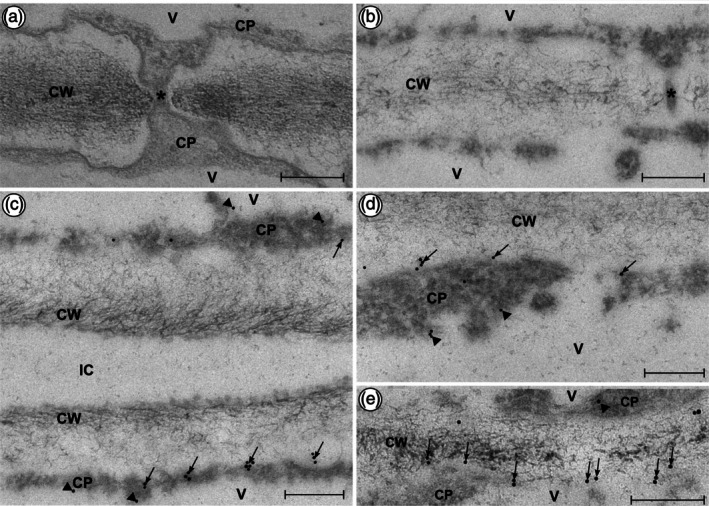
Immunolabeling of AGPs detected with JIM13 primary antibodies and 10 nm gold‐conjugated secondary antibodies in epidermal cells of *Anthoceros agrestis* gametophyte thalli observed by TEM. (a) For better orientation, a micrograph of a sample conventionally fixed with glutaraldehyde and osmium tetroxide and embedded in Spurr's Resin is shown. The asterisk marks a plasmodesmos. (b) Negative control section fixed for immunocytochemistry, treated with the secondary antibody only. A plasmodesmos is marked with an asterisk. No nonspecific immunolabels are present. (c–e) Detail images of samples fixed for immunocytochemistry including JIM13. (c, d) Single gold labels and small clusters of gold particles were regularly found at the plasma membrane (black arrows), but also at the tonoplast membrane of the vacuole (arrowheads). (e) Younger, still developing cell walls. Labeling occurs in the area of the plasma membrane (black arrows) and the tonoplast (black arrowheads), but also in the central fibrillar wall layers. Scale bars: 200 nm, CP, cytoplasm; CW, cell wall; IC, intercellular space; V, vacuole.

The JIM13 antibody was also used to detect AGPs immunocytochemically in *Anthoceros* tissue (Figure 5). For comparison and better orientation, Figure 5(a) shows a detail of a plasmodesmos (asterisk) traversing the cell wall (CW) of *Anthoceros* thallus cells with complete structural preservation of, for example, the bilayered plasma membrane due to conventional fixation which, however, hinders immunoreactivity. To estimate the specificity of the immunolabeling, TEM micrographs in Figure 5(c–e), showing thalli (fixed for immunocytochemistry and treated with the primary antibody JIM 13 and gold‐conjugated secondary antibodies), were compared with corresponding negative controls lacking JIM13 treatment (as an example see Figure 5b). Specific AGP labeling frequently appeared at the cell wall/plasma membrane interface (black arrows in Figure 5c–e), which is a typical location of GPI‐anchored AGPs. Furthermore, some labeling appeared in the central fibrillar layers of young and still developing cell walls (Figure 5d). Additionally, labeling of the tonoplast was observed (Figure 5c–e, arrowheads).

## DISCUSSION

The step of terrestrialization, about 470 million years ago, with its significant change in environmental conditions forced plants to develop coping strategies (Domozych & Bagdan, 2022). Comparative studies on extant bryophytes and their direct sister group, the tracheophytes, enable today's research to gain more insights into strategies which had already evolved in their MRCA (Bechteler et al., 2023). Cell wall composition was most likely one of the features which had been strongly influenced during this process. There is a lack in knowledge on bryophyte AGPs when it comes to analytical characterization exceeding immunocytochemical screening. To fill this gap, *Anthoceros* and *Physcomitrium* were analyzed in this study, focusing on in‐depth analysis of the AGPs which represent a major class of signaling glycoproteins in plant cell walls. The use of Yariv's reagent in the AGP isolation process results in purified AGP fractions with the advantage that all AGP‐like molecules which contain at least seven 1,3‐linked Galp residues are precipitated (Kitazawa et al., 2013; Paulsen et al., 2014). Side‐chain decorations were not described to influence the interaction with Yariv's reagent.

### Differing AGP contents in bryophytes seem to be lineage‐specific and dependent on culture conditions

Bryophytes have been studied in the past with regards to AGP contents. While most studies were based on rapid screening methods such as gel diffusion assays or antibody reactivities (Basile & Basile, 1987; Berry et al., 2016; Clarke et al., 1978; Kremer et al., 2004; Lee et al., 2005; Ligrone et al., 2002), only few studies isolated and characterized AGPs from mosses (three different species; Bartels et al., 2017; Fu et al., 2007; Lee et al., 2005) or liverworts (only *M. polymorpha*; Happ & Classen, 2019). With this study, we completed the available structural data on bryophyte AGPs with the currently unstudied bryophyte group of hornworts.

The setaphytes studied here (*Physcomitrium*, 0.09% AGP yield) and before show comparable yields to each other and to ferns (0.06–0.19%; Bartels & Classen, 2017; Bartels et al., 2017; Happ & Classen, 2019; Mueller et al., 2023). *Anthoceros* has much higher AGP contents, both in *in vitro* whole plant culture (0.31%, Figure S3) and suspension cell culture (0.57%). While the latter is in line with the observation that plant material derived from liquid cultures contain higher amounts also in *Physcomitrium* (0.23%; Bartels et al., 2017), the amount of AGPs from *Anthoceros* is elevated in both culture conditions.

### Bryophyte AGPs possess the conserved galactan structure known from tracheophytes

The polysaccharide part of the AGP of *Anthoceros* mainly consists of Ara, Gal and 3‐*O*‐MeRha, which together make up over 90%. In contrast, these monosaccharides account for only 70% in *Physcomitrium* AGP due to a high content of Glc. The high Glc content confirms the results obtained by other authors (Fu et al., 2007), but is contradictory to other studies, in which *Physcomitrium* AGP contained only small amounts of Glc (Bartels et al., 2017; Lee et al., 2005). Similar to our study, linkage‐type analyses revealed dominance of 1,4‐Glc in α‐anomeric linkage, indicating that Glc is principally starch, that was resistant against starch removal by DMSO and persisted through the precipitation with βGlcY (Fu et al., 2007). This result is unexpected, as normally βGlcY yields pure AGP fractions and no report is known of covalent linkages of AGPs to starch. Preliminary results from enzyme‐assisted release of glucose with following quantification using a glucose‐oxidase test with colorimetric detection also hinted toward presence of starch in *Physcomitrium* (Figure S2).

The typical galactan core of angiosperms, consisting of 1,3‐, 1,6‐, and 1,3,6‐linked Gal*p*, was also present in bryophyte AGPs. While angiosperm AGPs often have elaborate 1,6‐linked unbranched galactan side chains, these were present in lower amounts in bryophyte AGPs. This was also confirmed by the ELISA results with the antibody KM1, which did not bind to the *Anthoceros* AGP and showed only weak interaction with *Physcomitrium* AGP. The epitope of this antibody is a structure rich in 1,6‐Gal*p* linkages (Classen et al., 2004; Ruprecht et al., 2017). In addition, these low amounts seem to be characteristic for other AGPs of spore‐producing land plants and indicate a high degree of side‐chain branching of these AGPs (Bartels et al., 2017; Bartels & Classen, 2017; Happ & Classen, 2019; Lee et al., 2005; Mueller et al., 2023). Only the investigated peatmoss *Sphagnum* showed 1,6‐Gal values in the range of seed plants (Bartels et al., 2017; Thude & Classen, 2005). The typical AGP galactan structure has also been proven by search for the enzymatic toolkit responsible for the biosynthesis of the core galactan and its attachment to the protein backbone: Genes for prolyl‐4‐hydroxylases, Hyp‐*O*‐galactosyltransferases, β‐1,3‐galactosyltransferases, and β‐1,6‐galactosyltransferases (Silva et al., 2020) are present in the investigated bryophyte genomes. There are slightly higher homolog numbers in *Physcomitrium*, which could be a result of the described whole genome duplication (WGD) events (Rensing et al., 2020) with following sub‐ and neofunctionalization of several genes. In contrast, all hornworts lacked a WGD event during their evolution (Schafran et al., 2025)—for most liverworts, the same applies (except for a recent WGD in *Calypogeia fissa*; Castanedo et al., 2025).

### AGP side‐chain characteristics known from other non‐seed plant lineages were likely present in the common ancestor of all land plants

In angiosperm AGPs, Ara*f* is the dominant terminal residue, accompanied by terminal (4‐*O*‐Me)Glc*p*A, Gal*p*, and sometimes Fuc*p* (Tryfona et al., 2012). Ara*f* is also dominant in *Anthoceros* and *Marchantia* AGPs (Happ & Classen, 2019), but not in *Physcomitrium*. There, the unusual terminal (3‐*O*‐Me)Rha*p* represents most end groups (see also Lee et al., 2005). Results obtained by ELISA with the antibody JIM13, which is commonly used to detect AGPs in plant tissue (e.g., Knox et al., 1991) confirm our findings. Even though the exact epitope is still unknown, the trisaccharide β‐d‐Glc*p*A‐(1 → 3)‐α‐d‐Gal*p*A‐(1 → 2)‐α‐l‐Rha has been shown to bind this antibody (Yates et al., 1996). Furthermore, a rhamnogalactan‐protein from *Spirogyra* algae (Zygnematophyceae) reacted intensely with JIM13 (Pfeifer, Utermöhlen, et al., 2022). The differential affinity of *Physcomitrium* (higher) and *Anthoceros* (lower) supports that terminal Rha is involved in antibody recognition. Terminal 3‐*O*‐MeRha*p* has previously been found in different cell wall components (including AGPs) of algae, bryophytes, ferns, and gymnosperms (Akiyama et al., 1987; Bartels et al., 2017; Bartels & Classen, 2017; Baumann et al., 2021; Happ & Classen, 2019; Mueller et al., 2023; Ogawa et al., 1997; Pfeifer, Utermöhlen, et al., 2022; Popper & Fry, 2003), but it seems to be lost during the evolution of angiosperms since it has never been detected in any cell wall component of angiosperm species. To date, rhamnosyltransferases acting on AGPs are completely unknown. Thus, bryophytes are an interesting genomic resource for searches for these enzymes. No enzyme modifying Rha residues with methyl ethers is described. Therefore, a further challenge will be to search for the corresponding methyltransferases. In *Arabidopsis*, the enzymes AGM1 + 2, belonging to the DUF579 family, are responsible for the methylation of terminal GlcA in AGPs (Temple et al., 2019). Bryophytes did not contain methylated GlcA, and also close homologs of AGM1 + 2 are lacking in their genomes. Other members of the DUF579 family are present (Data S2) and might be responsible for the methylation of Rha in bryophyte AGPs.

*Anthoceros* and *Physcomitrium* AGPs contain small amounts of GlcA, which are added to Gal chains by GlcATs of CAZy family GT14 (Dilokpimol & Geshi, 2014; Knoch et al., 2013) for which genes are also present in genomes of the investigated hornworts, *Marchantia* and *Physcomitrium*.

Linear arabinose structures, such as 1,5‐Ara*f*, are commonly found as glycan decorations in angiosperm AGPs (Classen et al., 2000; Thude & Classen, 2005; Wack et al., 2005). The *Physcomitrium* data in this study, as well as other studies on moss (Bartels et al., 2017; Fu et al., 2007; Lee et al., 2005) or *Equisetum* (horsetail; Bartels & Classen, 2017) AGPs confirm the presence of 1,5‐Ara*f* also in other land plant lineages. Our study shows a difference in the two bryophyte species *Physcomitrium* and *Anthoceros* with the presence of 1,5‐Ara*f* specifically in *Physcomitrium* and absence in *Anthoceros*. This was also confirmed by their different affinity to LM6 by ELISA. The AGP of *Anthoceros* revealed a 1,3‐Ara*f* linkage type, similar to *Marchantia* and *Polytrichastrum* AGPs (Bartels et al., 2017; Happ & Classen, 2019). Arabinose‐rich cell wall structures have been hypothesized to contribute to the drought tolerance of several “resurrection plant” species (Moore et al., 2013). One of the key challenges for terrestrial life was drought tolerance. It was shown that *P. patens* is able to tolerate severe drought stress (Frank et al., 2005). The presence of polymeric arabinoses, either as pure arabinans or attached to other cell wall components, including AGPs, could therefore be understood as one coping strategy. Comparison of these results with other data (see Mueller et al., 2023) reveals a diversification of arabinose linkage types (1,2‐, 1,3‐ or 1,5‐Ara*f*) in different land plant lineages. The functions of this fine‐tuning are still to be identified. Up to now, only one AraT (RAY1, member of family GT77) working on AGPs has been described (Gille et al., 2013). One single close homolog of RAY1 has been detected in all investigated bryophyte genomes and may be responsible for arabinosylation of bryophyte AGPs. Although *Arabidopsis* knockout mutants of RAY1 showed reduced Ara content, the influence was strongest for 1,3‐linked Ara compared to other linkage types (Gille et al., 2013). The fact that bryophyte arabinose linkages were different shows that it is likely that more—yet unknown—AraTs are involved in arabinosylation of AGPs.

### Bryophytes have a reduced set of classical AGP protein backbones

All hornwort genomes as well as those of *Marchantia* and *Physcomitrium* contain sequences coding for AGP protein backbones. The protein backbones of AGPs are rich in the amino acids hydroxyproline/proline (Hyp/Pro), alanine (Ala), serine (Ser) and threonine (Thr), which are regularly arranged as Ala‐Pro, Ser‐Pro, and Thr‐Pro (for review, see Ma et al., 2018).

Classical AGPs occur with and without GPI‐anchors, which link AGPs to the plasma membrane and facilitate co‐location of signaling partners in lipid microdomains (Konrad & Ott, 2015). Our results reveal an impressive difference between hornworts and the setaphytes: GPI‐AGPs are mostly missing in hornwort genomes (10 species without sequence, 2 species with one sequence), whereas *Marchantia* and *Physcomitrium* genomes contain 20 and 16 sequences, respectively. This is comparable to *Arabidopsis* with 17 sequences coding for GPI‐AGPs (Johnson, Cassin, Lonsdale, Bacic, et al., 2017). The low content of GPI‐AGPs in hornworts compared to liverworts and mosses and also lycophytes, ferns, gymno‐ and angiosperms has also been shown for transcriptomes (Johnson, Cassin, Lonsdale, Ka‐Shu Wong, et al., 2017). The same trend is following for AGPs without GPI‐anchor: less sequences are detected in the hornwort genomes (in mean 2.9 sequences; range 0–7) compared with *Marchantia* (9 sequences) and *Physcomitrium* (18 sequences), the latter being in the range of *Arabidopsis* (12 sequences). To date, monophyly of hornworts, liverworts and mosses with hornworts as sister to the setaphytes is generally accepted (Donoghue et al., 2021; Puttick et al., 2018; Su et al., 2021). As genes for GPI‐AGPs have already been detected in transcriptomes of green algae (Johnson, Cassin, Lonsdale, Bacic, et al., 2017) and have been present in the common ancestor of all land plants, it seems that they have been reduced and mostly lost in extant hornworts. It is currently unknown which exact functionality classical AGPs possess. Generally, most described functions of AGPs are based on molecular activities of the sugar chain attached to the protein (Ca^2+^‐binding *via* AGP‐GlcA residues, Lamport & Várnai, 2013; the AG chain AMOR which is relevant for pollen tube guidance, Su & Higashiyama, 2018; interaction of AGPs with plasma membrane localized receptor‐like kinases, Ma & Johnson, 2023). Lower amounts of GPI‐AGPs would mean a less pronounced AG‐surface directly attached to the plasma membrane. Whether this has functional implications on, for example, the capability to interact with cell wall polymers and wall ions (Tan et al., 2018) has to be investigated in the future.

AGP protein backbones are characterized by high heterogeneity. Besides classical AGPs discussed above, hybrid AGPs combine AGP domains with sequences of other HRGPs (e.g., AGP motifs + extensin motifs) and chimeric AGPs are characterized by one or more additional functional protein domains (Ma et al., 2017). Their gene homologs are also present in at least some of the investigated members of all bryophyte taxa, with chimeric AGPs being clearly more abundant and widespread. Our biochemical data revealed a higher proportion of protein in the precipitated bryophyte AGPs (16–17%, *w/w*) in comparison to angiosperms, whereas the average amount of Hyp per protein was lower (1.5–1.9%, *w/w*). This indicates fewer glycosylation sites in the protein backbones. In light of our bioinformatic data, this supports the predominant presence of chimeric AGPs which contain additional (Hyp free) regions in their protein backbones beside the Hyp‐rich AG motifs.

### Chimeric AGPs seem to exhibit key AGP functions in bryophytes

Among these chimeric AGPs two types stand out in connection to the adaptation process to the terrestrial habitat: fasciclin‐like AGPs and xylogen‐like AGPs.

Fasciclin‐like AGPs contain a FAS1 (pfam02469) domain capable of binding multiple ligands and often involved in cell adhesion (Seifert, 2021). In some streptophyte algae, it has been shown that AGPs most likely function as key adhesion molecules (LoRicco et al., 2024; Palacio‐López et al., 2019). Fasciclin‐like AGPs are present in all investigated bryophyte genomes, and, therefore, they might have been important features for the ancestors of extant bryophytes to cope with life on land.

Besides adhesion, a huge challenge during terrestrialization was adaptation to water deficiency. In the clade of tracheophytes (lycophytes, ferns, and seed plants), long‐distance transport of water occurs in highly specialized tracheary elements. Historically, it was speculated that transporting tissue has been an innovation in tracheophytes. Today, it is known that simple conductive tissue is widespread among setaphytes, although occurrence and morphology are variable (Woudenberg et al., 2022). An involvement of AGPs in the development of water‐transporting tissue seems probable, as antibodies directed against AGP glycan epitopes bound to xylem elements of angiosperms (Bossy et al., 2009; Dolan et al., 1995; Goellner et al., 2013). Some of these antibodies also labeled water‐conducting cells of different liverworts and mosses (Ligrone et al., 2002). Especially the chimeric xylogen‐like AGPs are candidates possibly involved in the development of water‐conducting tissue. The chimeric AGP called “xylogen” is responsible for xylem development in cell cultures of the angiosperm *Zinnia* (Motose et al., 2004). Sequences of xylogen‐like AGPs were more abundant in *Marchantia* and *Physcomitrium* compared to the hornworts, which corresponds to the morphological finding that conductive tissues like water‐conducting cells are absent in hornworts (Ligrone et al., 2000; Woudenberg et al., 2022). On the other hand, our finding that sequences of xylogen‐like AGPs appear in hornwort, liverwort, and moss genomes supports the hypothesis that an evolutionary continuity is more likely than a strict gap between water‐conducting tissue of bryophytes and vascular plants (Woudenberg et al., 2022). Another observation was also that all organisms living in an aquatic habitat (*Azolla*, *Salvinia*, *Isoetes*) have generally lower numbers of xylogen‐like AGPs (Table S8). In this study, we were able to further classify the type of nsLTP domains included in the bryophyte xylogen‐like AGPs, which are highly congruent. Our findings suggest that xylogen‐like AGP evolution and evolution of nsLTP domains in general are deeply interconnected. This becomes evident when comparing our findings with the published data of Edstam et al. (2011). These authors proposed an evolutionary history of nsLTPs with D‐ and G‐type domains in the MRCA of all land plants while all streptophyte algae lack nsLTPs. We found 4 G‐type nsLTP domains in xylogen‐like AGPs in *Marchantia* corresponding to a total of 4 G‐type nsLTP sequences in *Marchantia* expressed sequence tags (ESTs, Edstam et al., 2011). Therefore, we hypothesize that all G‐type nsLTP sequences were initially chimeric AGP sequences, and a following diversification led to all other (nonchimeric) nsLTPs.

### Transmission electron microscopy (TEM) reveals antibody‐labeling of hornwort plasma membranes despite a lack of classical GPI‐anchored AGPs

In our study, AGP labeling was present at the plasma membrane/cell wall interface, a typical localization of GPI‐anchored AGPs, but also at the membrane of the vacuole, the tonoplast. In some immunohistochemical studies, JIM13 has already been used to detect AGPs in bryophytes (Berry et al., 2016; Kremer et al., 2004; Lee et al., 2005; Mansouri, 2012; Shibaya & Sugawara, 2009). In general, it is well‐established knowledge that AGPs are cell wall glycoproteins which can be plasma membrane bound or secreted into the extracellular matrix (Ma & Johnson, 2023), including mucilages (Płachno et al., 2020) or media of suspension cultures (Classen, 2007). In contrast, their subcellular distribution is largely unknown. Due to the detailed investigation on JIM13‐positive AGPs in *Physcomitrium* (Mansouri, 2012), we focused on *Anthoceros* in our study.

Comparable with the results of Mansouri (2012), our results demonstrated the presence of AGPs at the plasma membrane and the electron‐lucent wall layers close to it. As no classical GPI‐anchored AGPs were identified in *Anthoceros* (see above), chimeric AGPs (of which at least the xylogen‐like AGPs are GPI‐anchored) are possibly responsible for this labeling at the plasma membrane. Localization of proteins containing the nsLTP domain in *Physcomitrium* was investigated by Edstam et al. (2014). The authors found fluorescent‐tagged GPI‐anchored lipid transfer proteins (G‐type) located at the exterior side of the plasma membrane. As all hornwort xylogen‐like AGPs contain this type of domain, it is very likely that their cellular distribution is similar and can be labeled by JIM13. The median, fibrillar wall layers were labeled in young, still developing cell walls, but not in mature walls, which indicates a developmental modification of the AGP distribution. In both developmental stages, no AGPs could be detected at plasmodesmata or at the surrounding wall area, although it has been shown for *Arabidopsis thaliana* that AGPs play an essential role in plasmodesmal biogenesis (Okawa et al., 2023). It is conceivable, however, that the AGPs required for this function cannot be detected by the JIM13 antibody or differ between *Arabidopsis* and *Anthoceros*. Moreover, the two species differ drastically with respect to the plasmodesmal development (Wegner & Ehlers, 2024). We also observed an AGP labeling at the tonoplast. Occurrence of AGPs at the vacuole membrane has already been reported for some angiosperm plants (Olmos et al., 2017; Sala et al., 2017; Šamaj et al., 2000). In adventitious roots of tomato, the JIM13 epitope was localized at the tonoplast (Sala et al., 2017). In tobacco cell cultures, JIM13 AGP epitopes were located at the plasma membrane and also in the cytoplasm associated with the endoplasmic reticulum, the Golgi apparatus, vesicles, and the tonoplast (Olmos et al., 2017). The authors propose a role of AGPs as sodium carriers through vesicle trafficking from the plasma membrane to the tonoplast during salt stress. Herman and Lamb (1992) supported the hypothesis that AGPs can move from the plasma membrane to the tonoplast, since the AGP epitope was detected in the plasma membrane, in invaginations of the plasma membrane, in intracellular multivesicular bodies (= endosomes) and finally in partially degraded multivesicular bodies sequestered within the central vacuole. There might be still unknown functions of AGPs, especially connected with localization at the tonoplast.

## CONCLUSION

To date, only a few AGPs have been isolated from moss and liverwort cell walls. In this study, AGPs of a hornwort have been characterized and bioinformatic search for enzymes involved in AGP biosynthesis and for AGP backbone proteins has been performed for 12 hornwort genomes in comparison to *Marchantia* and *Physcomitrium* genomes. There were striking differences between the hornworts and the setaphytes, as sequences for AGP protein backbones were less abundant in hornworts compared with *Marchantia*, *Physcomitrium* and also *Arabidopsis*. GPI‐AGPs were completely missing in 10 of 12 hornwort genomes. Sequences encoding glycosyltransferases involved in AGP galactosylation were present in all bryophyte genomes, and the biochemical investigations also revealed presence of the typical AGP galactan framework in *Anthoceros* and *Physcomitrium*. This underlines that Hyp‐rich proteins highly glycosylated by branched galactans already occurred in the MRCA of all land plants. These were probably necessary for adaptation to life on land. This is supported by the presence of sequences encoding fasciclin‐like AGPs in all bryophyte genomes, which are described to play a role in adhesion, a fundamental prerequisite for plants conquering land. Furthermore, all bryophyte genomes except one contained sequences of xylogen‐like AGPs, indicating that—although bryophytes contain no xylem—one of the basal requirements for development of water‐conducting tissues is already present in all bryophyte taxa. This includes also hornworts which have not evolved morphologically differentiated conductive tissues. A special feature of bryophyte AGPs and highly abundant in *Physcomitrium* is terminal 3‐*O*‐methylrhamnose. This unusual monosaccharide also occurs in fern and some gymnosperm AGPs, but not in angiosperms. Identification of the corresponding rhamnosyl‐ and methytransferases is a challenging task for the future. In TEM, AGPs were detected in *Anthoceros* tissue at the plasma membrane/cell wall interface, as known for many angiosperms. Additionally, AGPs were present at the tonoplast, suggesting new functions of AGPs. Generation of AGP mutants in the model organisms *Marchantia* or *Physcomitrium* will help to elucidate functions of AGPs in bryophytes.

## MATERIAL AND METHODS

### Plant material and sampling

The hornwort *Anthoceros agrestis* paton (*Anthoceros*) was cultivated on agar medium in the Pharmaceutical Institute of Kiel University, Germany (Figure S3). Spores and fresh gametophyte material (Saxony strain formerly known as “Bonn”) were kind gifts of Péter Szövényi (University of Zurich). For spore germination and gametophyte culture on BCD medium (Cove et al., 2009), the protocol of Szövényi et al. (2015) was used. *Anthoceros agrestis* suspension culture material was a kind gift of Maike Petersen (University of Marburg). The material was cultivated in her lab according to Petersen (2003). The moss *P. patens* (hedw.) bruch & schimp (*Physcomitrium*) was grown in the greenhouse of the garden of the Pharmaceutical Institute. Fresh gametophyte material of *Physcomitrium* “Reute” strain was a kind gift of Stefan A. Rensing (University of Freiburg). All samples were cleaned with water and freeze‐dried.

### Isolation of water‐soluble cell wall fractions and arabinogalactan‐proteins (AGPs)

After freeze‐drying and grinding of the plant material, it was pre‐extracted two times (2 and 21 h) with acetone–water (70%, *V/V*) in a ratio of 1:10 (*w/V*) for *Anthoceros* and 1:20 (*w/V*) for *Physcomitrium*. The air‐dried plant residue was extracted with double‐distilled water (ddH_2_O) under constant stirring for 21 h at 4°C in a 1:50 (*w/V*) ratio for *Anthoceros* and 1:20 (*w/V*) for *Physcomitrium*. Afterwards, the aqueous extract was separated from the insoluble plant material in the case of *Anthoceros* by centrifugation (19 000 ***g***, 4°C, 20 min) or through a tincture press for *Physcomitrium*. The insoluble plant pellet was freeze‐dried and used for further extractions (see below).

Proteins in the aqueous extract were removed by heating to 90–95°C for 10 min with following centrifugation (20 min, 4°C, ≥ 4122 ***g***). The aqueous extract was then concentrated to approx. one‐tenth and precipitated with ethanol (final concentration: 80%, *V*/*V*) at 4°C, overnight. The resulting precipitate was separated by centrifugation (4°C, 19 000 ***g***, 20 min) and freeze‐dried afterwards (yielding AE).

AGPs were isolated from the AE with the β‐Glc‐Yariv reagent (βGlcY; 1 mg mL^−1^). The precipitated AGP‐βGlcY‐complex was separated and purified to gain the aqueous AGP fraction according to the procedure of Classen et al. (2005) and Mueller et al. (2023).

### Analysis of monosaccharides

The determination of the neutral monosaccharide composition was performed by acid hydrolysis (2 mol L^−1^ TFA, 1 h, 121°C) with following derivatization to the acetylated alditols. These were measured *via* gas chromatography (GC) with flame ionization detection (FID) and mass spectrometry detection (MSD; GC + FID: 7890B; Agilent Technologies, Santa Clara, CA, USA; MSD: 5977B; Agilent Technologies; column: Optima‐225; Macherey‐Nagel, Düren, Germany; 25 m, 250 μm, 0.25 μm; helium flow rate: 1 mL min^−1^; split ratio 30:1). A temperature gradient was used for peak separation (initial temperature 200°C, subsequent holding time of 3 min; final temperature 243°C with a gradient of 2°C min^−1^). The methylated monosaccharide 3‐*O*‐MeRha was identified by retention time and mass spectrum (see Happ & Classen, 2019).

The uronic acid content (UA) was performed according to published literature (Blakeney et al., 1983; Blumenkrantz & Asboe‐Hansen, 1973) with modifications as described by Mueller et al. (2023). Therefore, the uronic acids were derivatized with *m*‐hydroxydiphenyl and qualified photometrically at 525 nm by using a linear calibration line of a 1:1 GalA and GlcA mixture.

### Reduction of uronic acids of AGPs

Following the workflow of Taylor and Conrad (1972) with modifications described in Mueller et al. (2023), the uronic acids in 10–20 mg AGP sample were carboxy‐reduced with 216 mg of *N*‐cyclohexyl‐*N*‐[2‐(N‐methylmorpholino)‐ethyl]‐carbodiimide‐4‐toluolsulfonate and deuterium‐labeled with sodium borodeuteride solutions (2.0 mL of 1 mol L^−1^; 2.5 mL of 2 mol L^−1^; 2.5 mL of 4 mol L^−1^) to yield the AGP_UR_‐fraction.

### Structural characterization of AGPs

Linkage types of monosaccharides were determined using the methodology of Mueller et al., 2023, which is a modified version of Harris et al. (1984).

To achieve permethylation, the samples were first treated alternately with potassium methylsulfinyl carbanion and iodomethane in dimethyl sulfoxide and subsequently derivatized to permethylated alditol acetates (PMAA). These PMAAs were analyzed by gas –liquid chromatography‐mass spectrometry (see “Analysis of monosaccharides”; column: Optima‐1701, 25 m, 250 μm, 0.25 μm; helium flow rate: 1 mol L^−1^; initial temperature: 170 °C; hold time 2 min; rate 1°C min^−1^ until 210°C; rate: 30°C min^−1^ until 250°C; final hold time 10 min).

### Determination of hydroxyproline and protein content

The protein content (nitrogen content × 6.25; Kjeldahl, 1883) was determined by elemental analyses in the Chemistry Department of Kiel University, Kiel, Germany (HEKAtech CHNS Analyzer, Wegberg, Germany). Quantification of Hyp was performed photometrically at 558 nm according to Stegemann and Stalder (1967) with slight modifications (see Mueller et al., 2023). After acid hydrolysis (6 mol L^−1^, 22 h, 110°C), the Hyp content was coupled with 4‐dimethylaminobenzaldehyde and determined by linear regression analysis (Standard: 4‐hydroxy‐l‐proline).

### Indirect enzyme‐linked immunosorbent assay (ELISA)

ELISA experiments were performed as described in Mueller et al. (2023). The aqueous AGP solutions were used in the following concentrations: 12.5 , 25, and 50 μg mL^−11^. The primary antibodies (JIM13, LM2, LM6; Kerafast, Inc., Boston; USA; for KM1, see Classen et al., 2004) were diluted 1:20 (*V/V*). The secondary antibodies coupled with alkaline phosphatase (all from Sigma‐Aldrich Chemie GmbH, Taufkirchen, Germany), against rat IgG or mouse IgG (only for KM1) were used in a dilution of 1:500 (*V/V*) in phosphate‐buffered saline (PBS; pH 7.4). Epitopes of the primary antibodies and key references are listed in Table S3.

### Gel diffusion assay

Cavities were stamped into an agarose gel (Tris–HCl, 10 mmol L^−1^; CaCl_2_, 1 mmol L^−1^; NaCl, 0.9% *w/V*; agarose, 1% *w/V*) and loaded with 20 μL of a dilution of each AE sample (100 mg mL^−1^) or βGlcY (1 mg mL^−1^), respectively. Red precipitation bands appeared in positive samples after diffusion overnight in the dark.

### Plant culture and immunocytochemical transmission electron microscopy (TEM)

For TEM, *A. agrestis* (‘Saxony’ strain) plants were grown on soil in a green house. Gametophyte thallus fragments were cut and embedded in 2% low‐gelling Agarose (type VII, Sigma‐Aldrich, Steinheim, Germany). After fixing for 2 h each, at room temperature and on ice, in 1% (*w/V*) paraformaldehyde and 0.25% (*V/V*) glutaraldehyde in 50 mmol mL^−1^ sodium phosphate buffer; pH 7.2, and afterwards washing in buffer and ddH_2_O, the samples were contrasted for 2 h on ice in 0.5% uranyl acetate. Following dehydration in a graded ethanol series on ice (30, 50, 70, 90%), tissues were transferred into LR White hard grade (Agar Scientific, Stansted, UK), embedded in closed gelatin capsules and polymerized for 24 h at 50°C. Ultra‐thin sections (80 nm thick) were cut on a Reichert Om U2 ultramicrotome (Leica Microsystems GmbH, Wetzlar, Germany) and mounted on formvar‐coated single‐slot gold grids.

For immunolabeling, the sections were blocked for 1 h with 5% BSA in 100 mmol mL^−1^ PBS (pH 7.2), incubated with the primary antibody JIM13 (Table S1), diluted 1:20 in 0.5% BSA in PBS for 3 h, and treated with goat‐anti‐rat IgG (H + L)—10 nm gold conjugate (Cytodiagnostics, Burlington, Canada), diluted 1:50 in 0.5% BSA in PBS for 1 h. Washing with PBS was done after both antibody steps. Finally, the sections were treated with 3% glutaraldehyde for 10 min, washed again with ddH_2_O, and contrasted for 12 min each with 2% uranyl acetate and lead citrate (Reynolds, 1963). Analyses were performed with an EM912AB transmission electron microscope (Zeiss, Oberkochen, Germany) at 120 kV acceleration voltage under zero‐loss energy filtering conditions. A 2 k × 2 k dual‐speed slow‐scan CCD camera (SharpEye, TRS, Moorenweis, Germany) and the iTEM software package (OSIS) were used for image recording.

### Bioinformatic search for AGP backbone sequences and genes involved in AGP biosynthesis

Twelve hornwort (Schafran et al., 2025), one liverwort (Bowman et al., 2017), and one moss (Lang et al., 2018; Rensing et al., 2008) genomes were searched for AGP protein backbones with the workflow described in Mueller et al. (2023). In brief, protein sequences containing a putative N‐terminal signal peptide were initially identified through querying the SignalP 5.0 webserver (Almagro Armenteros et al., 2019). Those were then classified through the R‐implemented maab‐pipeline (Dragićević et al., 2020; Johnson, Cassin, Lonsdale, Bacic, et al., 2017). Small (<90 amino acids), highly similar (≥95%), and sequences containing any pfam domain (E‐value <1e‐5) were excluded from that analysis. GPI‐anchor prediction was performed with bigPI (Eisenhaber et al., 2003) and in case of ambiguities additionally with NetGPI 1.1 (Gíslason et al., 2021) and PredGPI (Pierleoni et al., 2008).

Chimeric AGP sequences including minimum one FAS1 (pfam02469), copper‐binding like (pfam02298) or nsLTP (pfam14368) domain were identified by querying the pfam database *via* the CDD webtool (Lu et al., 2020). To classify a sequence as chimeric AGP, minimum one region containing at least three dipeptides ([STAGV]P) separated by maximally six amino acids, should be present beside the known protein domain. After classification, the nsLTP domain was cut out with an addition of five amino acids at each end. Those trimmed regions were aligned using MAFFT in L‐INS‐i mode (version 7.490; Katoh & Standley, 2013) and manually inspected using Jalview (Waterhouse et al., 2009). Types of LTP domains based on spacing patterns between the eight conserved cysteins as well as presence of GPI‐anchors were classified according to Edstam et al. (2011).

The workflow for annotation of carbohydrate‐active enzymes (CAZy) was performed as described in Ali et al. (2025) and was identified using the dbCAN2 pipeline (Zhang et al., 2018) annotating them with three tools (HMMer against the CAZyme domain database, DIAMOND for BLASTp against the CAZyme database and dbCAN‐sub for HMMer detection of putative CAZy substrates). All sequences classified as one GT or GH family by a minimum two tools were filtered and aligned using MAFFT (preferably L‐INS‐i mode, otherwise FFT‐NS‐I mode; Katoh & Standley, 2013) or FastTree2 (for GT31 family; Price et al., 2010). Sequences similar to DUF579 and P4Hs of *Arabidopsis* were identified using BLASTp (E‐value of e^−7^) and aligned using MAFFT.

For comparison, genomes of two angiosperms (*A. thaliana*, Lamesch et al., 2011; *Amborella trichopoda*, Albert et al., 2013), two gymnosperms (*Cycas panzhihuaensis*, Liu et al., 2022; *Picea abies*, Nystedt et al., 2013), two lycophytes (*Isoetes taiwaniensis*, Wickell et al., 2021; *Selaginella moellendorffii*, Banks et al., 2011) and three ferns (*Azolla filiculoides*, *Salvinia cucullata*, Li et al., 2018; *Ceratopteris richardii*, Marchant et al., 2022) were used. Phylogenetic trees were inferred with IQ‐TREE (version 1.6.12; Nguyen et al., 2015) with built‐in model finder and visualized with iTOL (Letunic & Bork, 2021).

## AUTHOR CONTRIBUTIONS

**Kim‐Kristine Mueller:** Conceptualization, data curation, formal analysis, resources, investigation, visualization, writing—original draft, writing—review and editing. **Lukas Pfeifer:** Conceptualization, data curation, formal analysis, resources, investigation, visualization, writing—original draft, writing—review and editing. **Linus Wegner:** Data curation, formal analysis, investigation, visualization, writing—review and editing. **Katrin Ehlers:** Conceptualization, funding acquisition, project administration, writing—review and editing. **Birgit Classen:** Conceptualization, methodology, funding acquisition, project administration, writing—original draft, writing—review and editing.

## CONFLICT OF INTEREST

The authors declare no conflicts of interest.

## Supporting information


**Table S1.** Neutral monosaccharide composition of water‐soluble macromolecules from *Anthoceros agrestis and Physcomitrium patens* in % (mol mol^−1^).
**Table S2.** Content of uronic acids in water‐soluble polysaccharides and AGPs of *Anthoceros agrestis and Physcomitrium patens* in % (w w^−1^).
**Table S3.** Antibodies directed against AGP glycan motifs used in this study.
**Table S4.** Homolog numbers of characterized AGP‐active enzymes as identified by phylogenetic genome analysis of 12 hornwort and two setaphyte genomes.
**Table S5.** Numbers of identified protein sequences for classical (+/− GPI‐anchor) and hybrid arabinogalactan–proteins.
**Table S6.** Numbers of identified protein sequences for chimeric arabinogalactan‐proteins.
**Table S7.** Detailed analysis of sequence characteristics in nonspecific lipid transfer protein domains within xylogen‐like AGPs of bryophytes.
**Table S8.** Detailed analysis of sequence characteristics in nonspecific lipid transfer protein domains within xylogen‐like AGPs of selected other embryophytes.
**Figure S1.** Gel diffusion assay of aqueous extracts from *Anthoceros agrestis*, *Marchantia polymorpha*, and *Physcomitrium patens* with βGlcY.
**Figure S2.** Starch analysis in *Physcomitrium patens* AGP.
**Figure S3.** Culture of *Anthoceros agrestis* on agar plates.
**Data S1.** Phylogenetic tree for prolyl‐4‐hydroxylases (P4Hs).
**Data S2.** Phylogenetic tree for DUF579 homologs.
**Data S3.** Phylogenetic tree for glycosyltransferase 14 (GT14) family members.
**Data S4.** Phylogenetic tree for glycosyltransferase 29 (GT29) family members.
**Data S5.** Phylogenetic tree for glycosyltransferase 31 (GT31) family members.
**Data S6.** Phylogenetic tree for glycosyltransferase 37 (GT37) family members.
**Data S7.** Phylogenetic tree for glycosyltransferase 77 (GT77) family members.
**Data S8.** Phylogenetic tree for glycosylhydrolase 3 (GH3) family members.
**Data S9.** Phylogenetic tree for glycosylhydrolase 27 (GH27) family members.
**Data S10.** Phylogenetic tree for glycosylhydrolase 35 (GH35) family members.
**Data S11.** Phylogenetic tree for glycosylhydrolase 43 (GH43) family members.
**Data S12.** Phylogenetic tree for glycosylhydrolase 79 (GH79) family members.

## Data Availability

All relevant experimental data can be found within the manuscript and its supporting materials.
