# Supplementary material for: New insights into bryophyte arabinogalactan‐proteins from a hornwort and a moss model organism
Source: Plant J. 2025 Jun 30;123(1):e70312. doi: 10.1111/tpj.70312 (PMC12209756; doi:10.1111/tpj.70312)
Supplement: Supplementary file 1 — Table S1. Neutral monosaccharide composition of water‐soluble macromolecules from Anthoceros agrestis and Physcomitrium patens in % (mol mol−1). Table S2. Content of uronic acids in water‐soluble polysaccharides and AGPs of Anthoceros agrestis and Physcomitrium patens in % (w w−1). Table S3. Antibodies directed against AGP glycan motifs used in this study. Table S4. Homolog numbers of characterized AGP‐active enzymes as identified by phylogenetic genome analysis of 12 hornwort and two setaphyte genomes. Table S5. Numbers of identified protein sequences for classical (+/− GPI‐anchor) and hybrid arabinogalactan–proteins. Table S6. Numbers of identified protein sequences for chimeric arabinogalactan‐proteins. Table S7. Detailed analysis of sequence characteristics in nonspecific lipid transfer protein domains within xylogen‐like AGPs of bryophytes. Table S8. Detailed analysis of sequence characteristics in nonspecific lipid transfer protein domains within xylogen‐like AGPs of selected other embryophytes. Figure S1. Gel diffusion assay of aqueous extracts from Anthoceros agrestis, Marchantia polymorpha, and Physcomitrium patens with βGlcY. Figure S2. Starch analysis in Physcomitrium patens AGP. Figure S3. Culture of Anthoceros agrestis on agar plates. Data S1. Phylogenetic tree for prolyl‐4‐hydroxylases (P4Hs). Data S2. Phylogenetic tree for DUF579 homologs. Data S3. Phylogenetic tree for glycosyltransferase 14 (GT14) family members. Data S4. Phylogenetic tree for glycosyltransferase 29 (GT29) family members. Data S5. Phylogenetic tree for glycosyltransferase 31 (GT31) family members. Data S6. Phylogenetic tree for glycosyltransferase 37 (GT37) family members. Data S7. Phylogenetic tree for glycosyltransferase 77 (GT77) family members. Data S8. Phylogenetic tree for glycosylhydrolase 3 (GH3) family members. Data S9. Phylogenetic tree for glycosylhydrolase 27 (GH27) family members. Data S10. Phylogenetic tree for glycosylhydrolase 35 (GH35) family members. Data S11. Phyl [file TPJ-123-0-s001.pdf]

Supplementary data to:

New insights into bryophyte arabinogalactan-proteins from a hornwort and a moss model organism

Kim-Kristine Mueller[1†], Lukas Pfeifer[1†], Linus Wegner[2], Katrin Ehlers[2] and Birgit Classen[1*]

[1] *Pharmaceutical Institute, Department of Pharmaceutical Biology, Christian-Albrechts-University of Kiel, Gutenbergstraße 76, 24118 Kiel, Germany*
[2] *Institute of Botany, Justus Liebig University, Heinrich-Buff-Ring 38, 35392 Giessen, Germany*

[†] These authors contributed equally to this work.

[*] Corresponding author:

Birgit Classen, Pharmaceutical Institute, Department of Pharmaceutical Biology, Christian-Albrechts-University of Kiel, Gutenbergstr. 76, 24118 Kiel, Germany
Phone: +49-431-8801130
Fax: +49-431-8801352
e-mail: *bclassen@pharmazie.uni-kiel.de*

Table S1 Neutral monosaccharide composition of high molecular weight, water-soluble fractions (AE) from *Anthoceros agrestis* (plants and cell cultures) and *Physcomitrium patens* in % (mol mol^{-1}; n=3; tr: trace value < 1 %).

Neutral monosaccharide	*A. agrestis* plants	*A. agrestis* cell culture	*P. patens* plants
3-*O*-MeRha	1.3 ± 0.1	2.8 ± 0.0	2.7 ± 0.0
Rha	2.2 ± 0.1	1.7 ± 0.5	4.2 ± 0.0
Fuc	1.4 ± 0.1	5.2 ± 0.3	4.0 ± 0.1
Rib	tr	9.4 ± 0.8	2.1 ± 0.2
Ara	8.4 ± 0.1	28.5 ± 2.6	11.5 ± 0.2
Xyl	23.8 ± 0.1	11.1 ± 0.8	7.7 ± 0.1
Man	1.2 ± 0.0	7.1 ± 1.0	6.0 ± 0.2
Gal	42.2 ± 0.3	26.0 ± 0.5	14.2 ± 0.3
Glc	19.5 ± 0.2	8.2 ± 3.2	47.6 ± 0.8

Table S2 Content of uronic acids in high molecular weight, water-soluble fractions (AE) and AGPs from *Anthoceros agrestis* plants and cell cultures and *Physcomitrium patens*, in % (w w^{-1}).

	A. agrestis plants	*A. agrestis* cell culture	*P. patens* plants
AE	11.1	2.2	3.1
AGP	7.3	4.8	6.5

Table S3 Antibodies directed against AGP glycan motifs used in this study.

Antibody	Epitope	Key References
JIM13	AGP glycan, e.g. β-D-Glc*p*A-(1→3)-α-D-Gal*p*A-(1→2)-α-L-Rha	Pfeifer *et al.* (2022b); Yates *et al.* (1996)
KM1	(1→6)-β-D-Gal*p* units in AGs type II	Classen *et al.* (2004); Ruprecht *et al.* (2017)
LM2	(1→6)-β-D-Gal*p* units with terminal ß-D-Glc*p*A in AGPs	Ruprecht *et al.* (2017); Smallwood *et al.* (1996);
LM6	(1→5)-α-L-Ara*f* oligomers in arabinan or AGPs	Verhertbruggen *et al.* (2009)

Table S4 Homolog numbers of characterized AGP-active enzymes as identified by phylogenetic genome analysis of 12 hornwort and 2 setaphyte genomes.

**species**	**P4H1**	**P4H5**	**P4H13**	**GALT29A**	**GALT31A/KNS4 /UPEX1**	**GALT2-6**	**HPGT1-3**	**GALT9**	**GlcAT14A-E**	**FUTs (GT37)**	**RAY1 (GT77)**	**AGM1+2**	**RsAra*f***	**AGAL2-3**	**AtBGAL8**	**GH43**	**AtGUS2**
Leicosporoceros dussii	1	1	1	2	1	3	1	2	1	5	1	0	2	1	1	1	1
Anthoceros agrestis 'Bonn'	3	1	1	1	1	4	1	1	3	3	1	0	3	1	1	0	1
Anthoceros agrestis 'Oxford'	3	1	1	2	1	4	1	2	3	4	1	0	3	1	2	1	1
Anthoceros punctatus	1	1	1	2	1	3	1	2	3	4	1	0	3	1	2	1	1
Anthoceros fusiformis	1	1	1	2	1	2	1	2	3	4	1	0	3	1	2	2	1
Notothylas orbicularis	1	1	1	3	1	2	1	2	1	3	1	0	2	1	1	1	1
Paraphymatoceros pearsonii	1	1	2	3	1	3	1	2	1	3	1	0	3	1	2	1	1
Phaeoceros carolinianus	1	1	1	3	1	3	1	4	1	3	1	0	5	2	2	1	1
Phaeoceros sp.	1	1	1	4	1	3	1	2	1	3	1	0	6	3	2	1	1
Megaceros flagellaris	1	1	1	4	1	2	1	2	1	4	1	0	3	3	2	1	1
Phaeomegaceros chiloensis	1	1	1	3	1	2	1	2	1	4	1	0	5	3	0	1	1
Phymatoceros phymatodes	1	1	1	3	1	2	1	2	1	3	1	0	5	3	1	1	1
Marchantia polymorpha	0	1	1	4	2	2	1	1	1	3	1	0	5	1	3	1	3
Physcomitrium patens	2	2	2	2	4	3	2	2	7	6	1	0	2	1	4	2	4
**average hornworts**	**1.3**	**1.0**	**1.1**	**2.7**	**1.0**	**2.8**	**1.0**	**2.1**	**1.7**	**3.6**	**1.0**	**0.0**	**3.6**	**1.8**	**1.5**	**1.0**	**1.0**

Table S5 Numbers of identified protein sequences for classical (+/- GPI-anchor) and hybrid arabinogalactan-proteins. We used the motif and amino acid bias (MAAB) classification system of Johnson *et al*., 2017.

**species**	**GPI-AGP (class 1)**	**CL-EXT (class 2)**	**PRP (class 3)**	**Non-GPI-AGP (class 4)**	**Hybrid AGP (class 5-8)**	**GPI-EXT (class 9)**	**Hybrid EXT (class 10-14)**	**Hybrid PRP (class 15-18)**	**Shared bias (class 19-23)**	**Non-HRGPs (class 24)**
Leicosporoceros dussii	1.0	2.0	-	-	-	-	1.0	2.0	-	2.0
Anthoceros agrestis 'Bonn'	-	-	-	2.0	-	-	-	-	-	4.0
Anthoceros agrestis 'Oxford'	-	1.0	-	2.0	-	-	1.0	-	-	6.0
Anthoceros punctatus	1.0	1.0	-	1.0	-	-	1.0	-	-	5.0
Anthoceros fusiformis	-	-	-	1.0	-	-	1.0	1.0	-	3.0
Notothylas orbicularis	-	2.0	-	3.0	1.0	-	-	-	3.0	4.0
Paraphymatoceros pearsonii	-	1.0	-	4.0	-	-	-	-	1.0	1.0
Phaeoceros carolinianus	-	2.0	-	6.0	1.0	-	2.0	-	4.0	6.0
Phaeoceros sp.	-	4.0	-	7.0	2.0	-	2.0	1.0	3.0	8.0
Megaceros flagellaris	-	3.0	-	4.0	-	-	-	1.0	2.0	-
Phaeomegaceros chiloensis	-	3.0	-	2.0	-	-	1.0	-	1.0	1.0
Phymatoceros phymatodes	-	5.0	-	3.0	-	-	1.0	2.0	-	5.0
Marchantia polymorpha	20.0	1.0	-	9.0	1.0	-	3.0	1.0	2.0	13.0
Physcomitrium patens	16.0	-	-	18.0	2.0	-	-	-	3.0	1.0
**average hornworts**	**0.2**	**2.0**	**-**	**2.9**	**0.3**	**-**	**0.8**	**0.6**	**1.2**	**3.8**

Table S6 Numbers of identified protein sequences for chimeric arabinogalactan-proteins. We focused here on fasciclin-like, plastocyanin-like and xylogen-like AGPs.

**species**	**fasciclin-like AGPs (pfam02469)**	**plastocyanin-like AGPs (pfam02298)**	**xylogen-like AGPs (pfam14368)**
Leicosporoceros dussii	3	1	0
Anthoceros agrestis 'Bonn'	4	19	2
Anthoceros agrestis 'Oxford'	4	21	2
Anthoceros punctatus	2	12	2
Anthoceros fusiformis	2	5	2
Notothylas orbicularis	6	7	1
Paraphymatoceros pearsonii	3	9	1
Phaeoceros carolinianus	1	10	1
Phaeoceros sp.	3	23	1
Megaceros flagellaris	4	7	1
Phaeomegaceros chiloensis	2	12	1
Phymatoceros phymatodes	7	8	1
Marchantia polymorpha	9	21	3
Physcomitrium patens	5	13	6
**average hornworts**	**3.4**	**11.2**	**1.3**

Table S7 Detailed analysis of sequence characteristics in non-specific lipid transfer protein domains within xylogen-like AGPs of bryophytes. 12 hornwort genomes and two setaphyte genomes (*Physcomitrium patens*, Pp; *Marchantia polymorpha*, Mp) were searched. The column "type" uses the classification system of Edstam *et al*. (2011). For the two setaphytes some sequences contained two predicted domains which are here mentioned as "1st domain" and "2nd domain".

id	spacing patterns											GPI	type
AagrBONN_evm.model.Sc2ySwM_344.1345.2	C	9	C	14	CC	12	C-1-C	21	C	11	C	-	D/G
AagrBONN_evm.model.Sc2ySwM_368.1844.1	C	9	C	14	CC	12	C-1-C	22	C	8	C	✓	G
AnagrOXF.S3G187000.t1	C	9	C	14	CC	12	C-1-C	22	C	8	C	✓	G
AnagrOXF.S5G197600.t1	C	9	C	14	CC	12	C-1-C	21	C	11	C	✓	G
Anfus.S1G112900.t1	C	9	C	14	CC	12	C-1-C	21	C	9	C	-	D/G
Anfus.S3G155900.t1	C	9	C	14	CC	12	C-1-C	22	C	8	C	✓	G
Anpun.S1G391000.t1	C	9	C	14	CC	12	C-1-C	22	C	8	C	✓	G
Anpun.S3G522700.t1	C	9	C	14	CC	12	C-1-C	21	C	11	C	✓	G
Mefla.S3G388200.t1	C	9	C	14	CC	12	C-1-C	24	C	9	C	✓	G
Noorb.S3G288500.t1	C	9	C	14	CC	12	C-1-C	25	C	9	C	✓	G
Phcar.S4G164600.t1	C	9	C	14	CC	12	C-1-C	25	C	9	C	✓	G
Phchi.S1G653500.t1	C	9	C	14	CC	12	C-1-C	22	C	8	C	✓	G
Papea.S4G078400.t1	C	9	C	14	CC	12	C-1-C	22	C	9	C	-	D/G
Phphy.S3G353100.t1	C	9	C	14	CC	12	C-1-C	27	C	9	C	✓	G
Phsp.C5G108400.t1	C	9	C	14	CC	12	C-1-C	25	C	9	C	✓	G
Mp1g21600.1	C	9	C	14	CC	12	C-1-C	29	C	12	C	✓	G
Mp1g22240.1	C	9	C	14	CC	12	C-1-C	24	C	8	C	✓	G
Mp8g18180.1 / 1st domain	C	9	C	14	CC	12	C-1-C	27	C	8	C	✓	G
Mp8g18180.1 / 2nd domain	C	9	C	14	CC	12	C-1-C	21	C	8	C	✓	G
Pp3c11_11470V3.1.p	C	9	C	16	CC	12	C-1-C	23	C	8	C	✓	G
Pp3c11_8360V3.1.p	C	9	C	14	CC	12	C-1-C	23	C	8	C	✓	G
Pp3c14_3790V3.1.p	C	9	C	14	CC	12	C-1-C	27	C	8	C	✓	G
Pp3c1_31020V3.1.p / 1st domain	C	9	C	14	CC	12	C-1-C	27	C	8	C	✓	G
Pp3c1_31020V3.1.p / 2nd domain	C	9	C	15	CC	12	C-1-C	25	C	8	C	✓	G
Pp3c2_6280V3.1.p / 1st domain	C	9	C	15	CC	12	C-1-C	25	C	8	C	✓	G
Pp3c2_6280V3.1.p / 2nd domain	C	9	C	14	CC	12	C-1-C	27	C	8	C	✓	G
Pp3c7_20230V3.1.p	C	9	C	14	CC	12	C-1-C	23	C	8	C	✓	G

Table S8 Detailed analysis of sequence characteristics in non-specific lipid transfer protein domains within xylogen-like AGPs of selected other embryophytes. Two angiosperms (*Amborella trichopoda*, AMTR; *Arabidopsis thaliana*, AT), two gymnosperms (*Picea abies*, MA, *Cycas panzhihuaensis*, CYCAS), two lycophytes (*Isoetes taiwaniensis*, Itaiw; *Selaginella moellendorfii*, Smo) and three ferns (*Ceratopteris richardii*, Ceric; *Azolla filiculoides*, Azfi; *Salvinia cucullata*, Sacu) were analyzed. The column "type" uses the classification system of Edstam *et al*. (2011). Some sequences contained two predicted domains which are here mentioned as "1st domain" and "2nd domain".

id	spacing patterns											GPI	type
AMTR_s00002p00272100	C	9	C	20	CC	12	C-1-C	24	C	6	C	✓	G
AMTR_s00002p00272110	C	10	C	17	CC	12	C-1-C	24	C	8	C	✓	G
AMTR_s00002p00272120	C	9	C	14	CC	12	C-1-C	24	C	9	C	✓	G
AMTR_s00010p00204620	C	9	C	16	CC	12	C-1-C	24	C	9	C	✓	G
AT1G03103	C	9	C	14	CC	12	C-1-C	26	C	9	C	✓	G
AT1G05450	C	10	C	14	CC	12	C-1-C	24	C	8	C	✓	G
AT1G18280	C	6	C	13	CC	12	C-1-C	25	C	8	C	✓	G
AT1G27950	C	9	C	14	CC	12	C-1-C	29	C	9	C	✓	G
AT1G32280	C	10	C	17	CC	9	C-1-C	22	C	9	C	-	G/D
AT1G36150 (AtXYLP5)	C	9	C	16	CC	12	C-1-C	24	C	7	C	✓	G
AT1G73560	C	6	C	14	CC	12	C-1-C	25	C	8	C	✓	G
AT1G73890	C	9	C	14	CC	12	C-1-C	26	C	8	C	✓	G
AT2G13820 (AtXYP2)	C	9	C	16	CC	12	C-1-C	24	C	9	C	✓	G
AT2G44290 (AtXYLP9)	C	9	C	14	CC	12	C-1-C	26	C	8	C	✓	G
AT2G48130 (AtXYLP11)	C	9	C	14	CC	12	C-1-C	25	C	9	C	✓	G
AT2G48140	C	10	C	17	CC	12	C-1-C	25	C	8	C	✓	G
AT3G22600 (AtXYLP12)	C	9	C	14	CC	12	C-1-C	25	C	9	C	✓	G
AT3G22620	C	10	C	17	CC	12	C-1-C	24	C	8	C	✓	G
AT3G43720 (AtXYLP10)	C	9	C	18	CC	12	C-1-C	26	C	9	C	✓	G
AT4G08670 (AtXYLP3)	C	9	C	16	CC	12	C-1-C	24	C	8	C	✓	G
AT4G14805	C	9	C	17	CC	13	C-1-C	24	C	12	C	✓	G
AT5G09370 (AtXYLP4)	C	9	C	16	CC	12	C-1-C	23	C	9	C	✓	G
AT5G48490	C	9	C	15	CC	9	C-1-C	24	C	7	C	-	G/D
AT5G64080 (AtXYP1)	C	9	C	16	CC	12	C-1-C	24	C	9	C	✓	G
Azfi_s0011.g012661	C	9	C	14	CC	12	C-1-C	28	C	8	C	✓	G
Azfi_s0803.g087563	C	9	C	15	CC	12	C-1-C	26	C	9	C	✓	G
Azfi_s0803.g087563	C	9	C	15	CC	12	C-1-C	26	C	9	C	✓	G
Azfi_s0803.g087563	C	9	C	15	CC	12	C-1-C	26	C	9	C	✓	G
Ceric.01G082200.1.p	C	9	C	14	CC	12	C-1-C	27	C	8	C	✓	G
Ceric.04G032800.1.p	C	9	C	15	CC	14	C-1-C	22	C	10	C	✓	G
Ceric.05G095500.1.p / 1st domain	C	9	C	14	CC	12	C-1-C	24	C	9	C	✓	G
Ceric.05G095500.1.p / 2nd domain	C	9	C	14	CC	12	C-1-C	24	C	9	C	✓	G
Ceric.07G064700.1.p	C	10	C	17	CC	9	C-1-C	22	C	7	C	-	G/D
Ceric.12G092700.1.p	C	9	C	15	CC	14	C-1-C	24	C	10	C	✓	G
Ceric.14G098300.1.p	C	9	C	15	CC	14	C-1-C	24	C	10	C	✓	G
Ceric.32G069900.1.p	C	9	C	15	CC	14	C-1-C	24	C	9	C	✓	G
Ceric.35G030300.1.p	C	12	C	14	CC	12	C-1-C	24	C	9	C	✓	G
CYCAS_013046	C	9	C	14	CC	18	C-1-C	21	C	10	C	-	G/D
CYCAS_024350	C	9	C	11	CC	12	C-1-C	24	C	9	C	-	G/D
CYCAS_024351	C	9	C	16	CC	12	C-1-C	24	C	9	C	✓	G
CYCAS_024351	C	9	C	16	CC	12	C-1-C	25	C	9	C	✓	G

CYCAS_024353	C	9	C	16	CC	12	C-1-C	24	C	9	C	-	G/D
CYCAS_024354	C	9	C	18	CC	12	C-1-C	24	C	9	C	✓	G
CYCAS_024355	C	9	C	19	CC	12	C-1-C	24	C	9	C	-	G/D
CYCAS_024356	C	9	C	17	CC	12	C-1-C	24	C	9	C	✓	G
MA_10069449g0010	C	9	C	14	CC	12	C-1-C	26	C	8	C	-	G/D
MA_10432091g0010	C	9	C	15	CC	12	C-1-C	25	C	9	C	-	G/D
MA_113140g0010	C	9	C	19	CC	12	C-1-C	23	C	9	C	-	G/D
MA_138772g0010	C	9	C	16	CC	12	C-1-C	25	C	9	C	-	G/D
MA_169045g0010	C	9	C	16	CC	12	C-1-C	34	C	9	C	✓	G
MA_494876g0010	C	9	C	14	CC	12	C-1-C	24	C	9	C	-	G/D
MA_50191g0010	C	9	C	15	CC	12	C-1-C	24	C	9	C	✓	G
MA_697637g0010	C	9	C	17	CC	12	C-1-C	24	C	8	C	-	G/D
MA_71069g0010	C	9	C	14	CC	12	C-1-C	24	C	9	C	✓	G
MA_76307g0020	C	9	C	14	CC	12	C-1-C	24	C	9	C	✓	G
MA_8892965g0010	C	9	C	16	CC	12	C-1-C	24	C	9	C	-	G/D
Itaiw_v1_scaffold_52_t29397-RA	C	9	C	14	CC	12	C-1-C	26	C	8	C	✓	G
Sacu_v1.1_s0056.g014600	C	9	C	14	CC	12	C-1-C	28	C	8	C	✓	G
Sacu_v1.1_s0073.g017064 / 1st domain	C	9	C	15	CC	12	C-1-C	26	C	9	C	✓	G
Sacu_v1.1_s0073.g017064 / 2nd domain	C	9	C	15	CC	12	C-1-C	26	C	9	C	✓	G
Smo445118	C	10	C	13	CC	9	C-1-C	22	C	7	C	-	G/D

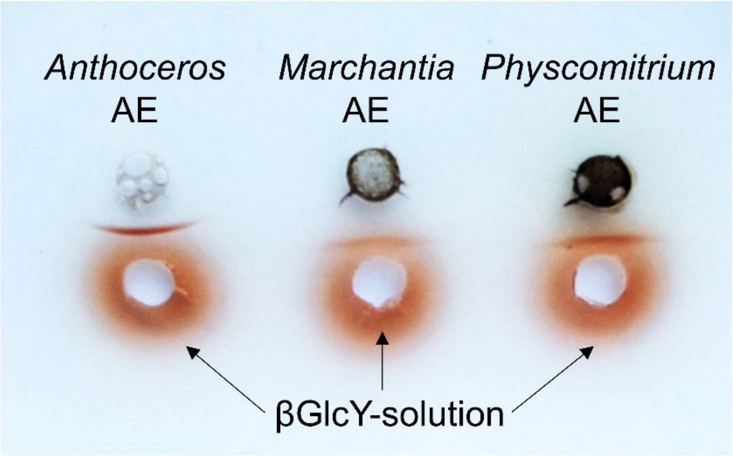

Figure S1. Gel diffusion assay of aqueous extracts from *Anthoceros agrestis*, *Marchantia polymorpha* and *Physcomitrium patens* (100 mg mL^{-1}) with βGlcY (1 mg mL^{-1}). The red precipitation line indicates presence of AGPs.

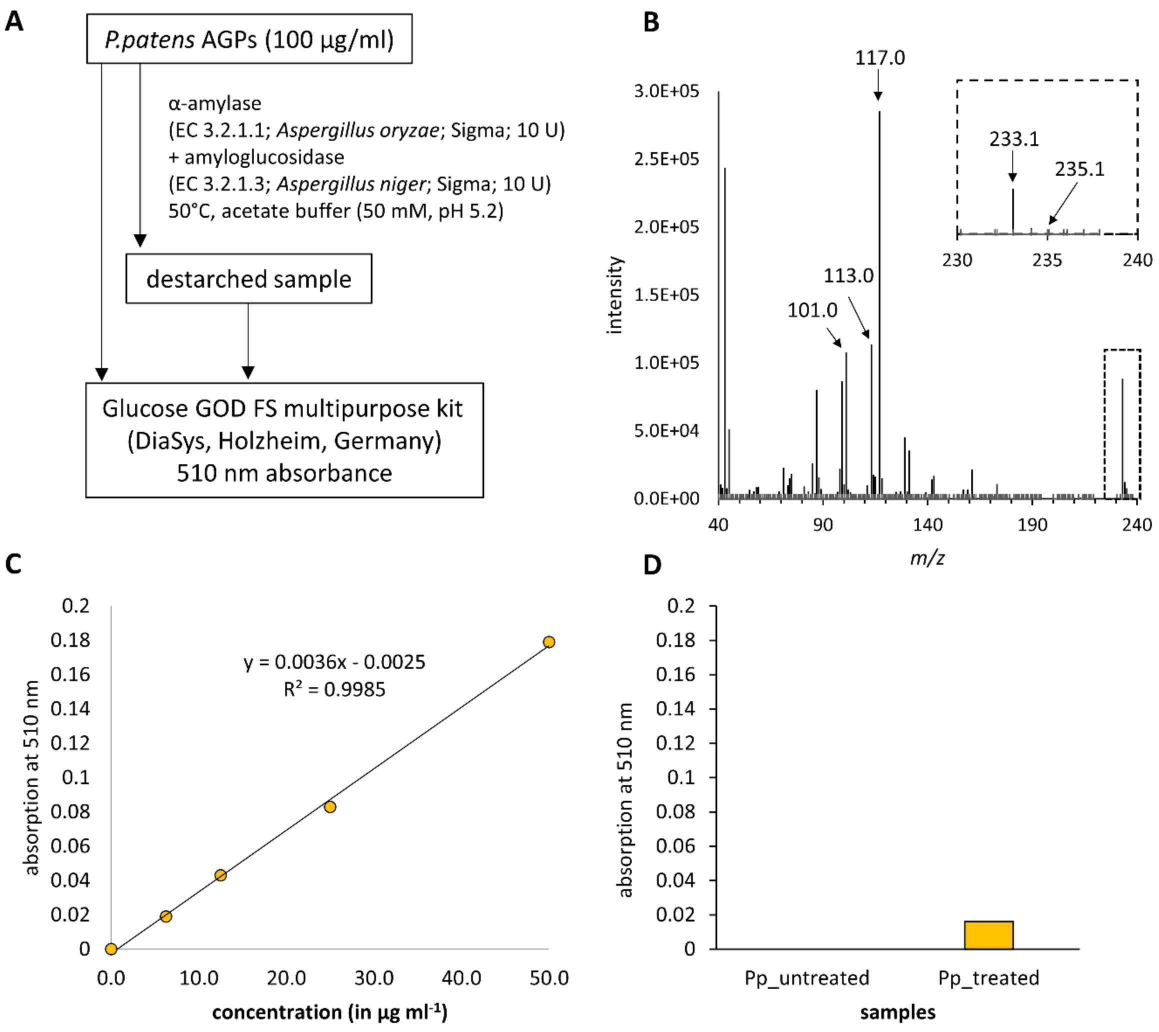

Figure S2. Starch analysis in *Physcomitrium patens* AGP. **A**: Workflow for enzymatic digestion and quantification of released glucose by a colorimetric glucose oxidase kit. **B**: Fragmentation pattern of partially methylated and acetylated 1,4-linked glucose in the mass spectrum of the uronic acid reduced sample of *P. patens*. The box highlights the region in which the two diagnostic primary ions m/z = 233 (1,4-linked Glc*p*) and m/z = 235 (1,4-linked Glc*p*A) are found. The extreme overrepresentation of m/z = 233.1 supports the presence of starch. **C**: calibration line for glucose determined with the glucose oxidase kit. **D**: Absorption of *P. patens* AGP before (Pp_untreated) and after (Pp_treated) digestion with α-amylase and amyloglucosidase. The calculated value for released glucose corresponds to approximately 11.2 % (w/w) in the AGP solution.

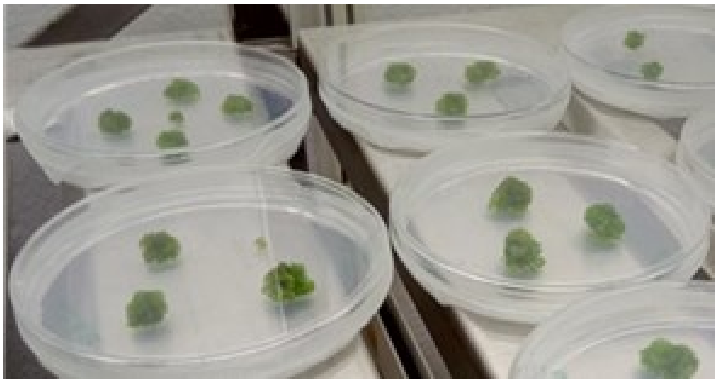

Figure S3. Culture of *Anthoceros agrestis* on agar in the Pharmaceutical Institute of Kiel University, Germany.

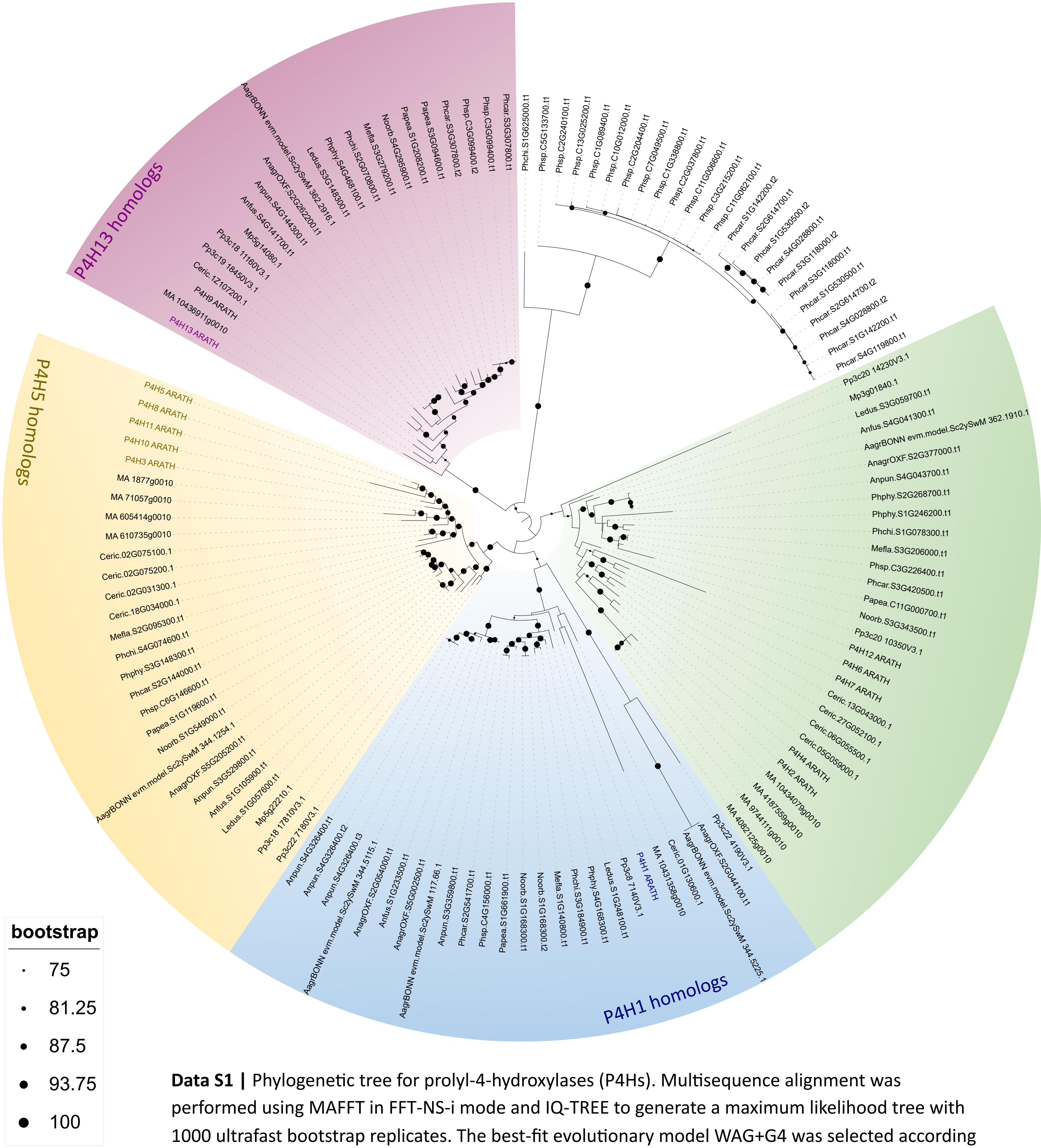

Data S1 | Phylogenetic tree for prolyl-4-hydroxylases (P4Hs). Multisequence alignment was performed using MAFFT in FFT-NS-i mode and IQ-TREE to generate a maximum likelihood tree with 1000 ultrafast bootstrap replicates. The best-fit evolutionary model WAG+G4 was selected according to Bayesian Information Criterion.

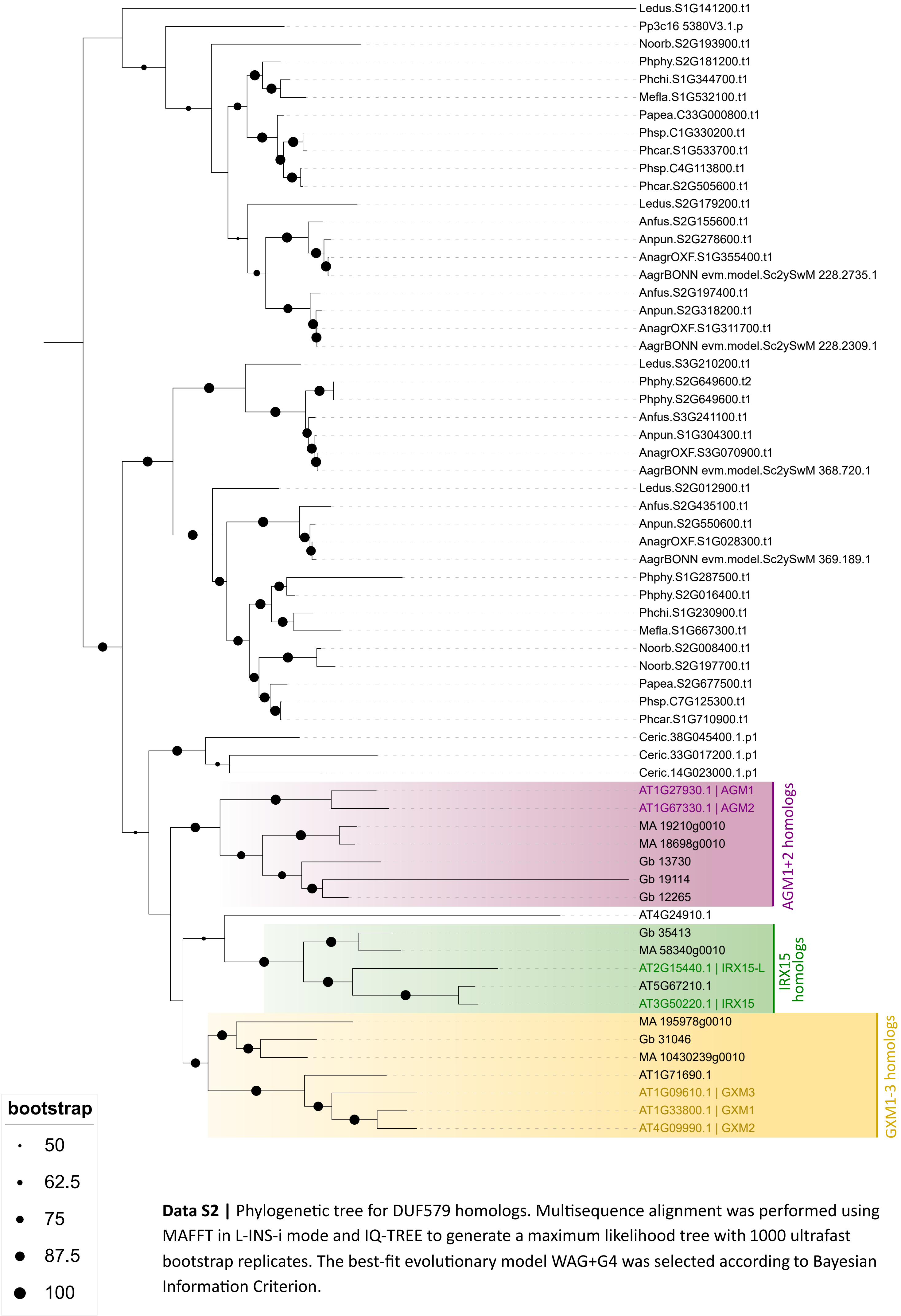

Data S2 | Phylogenetic tree for DUF579 homologs. Multisequence alignment was performed using MAFFT in L-INS-i mode and IQ-TREE to generate a maximum likelihood tree with 1000 ultrafast bootstrap replicates. The best-fit evolutionary model WAG+G4 was selected according to Bayesian Information Criterion.

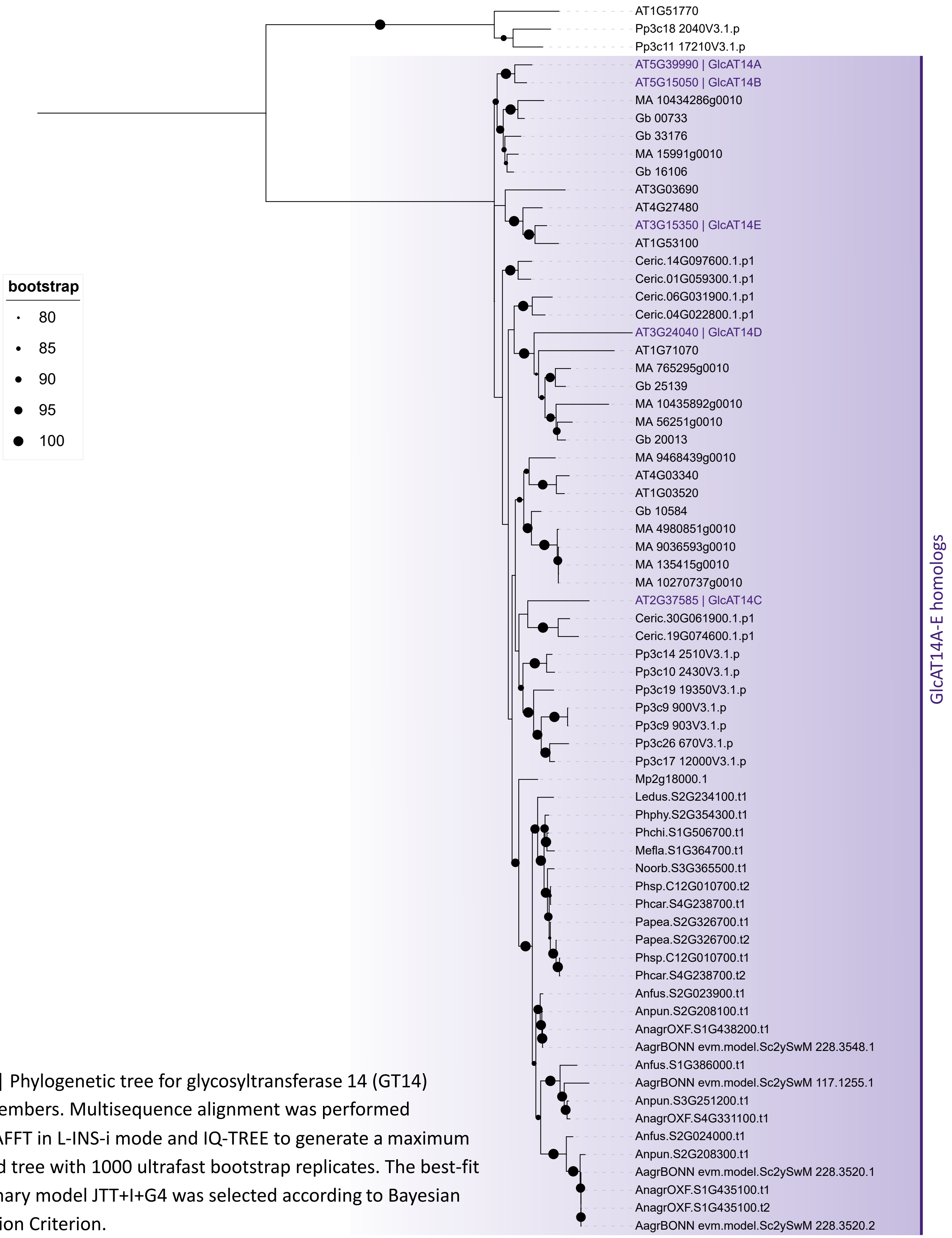

Data S3 | Phylogenetic tree for glycosyltransferase 14 (GT14) family members. Multisequence alignment was performed using MAFFT in L-INS-i mode and IQ-TREE to generate a maximum likelihood tree with 1000 ultrafast bootstrap replicates. The best-fit evolutionary model JTT+I+G4 was selected according to Bayesian Information Criterion.

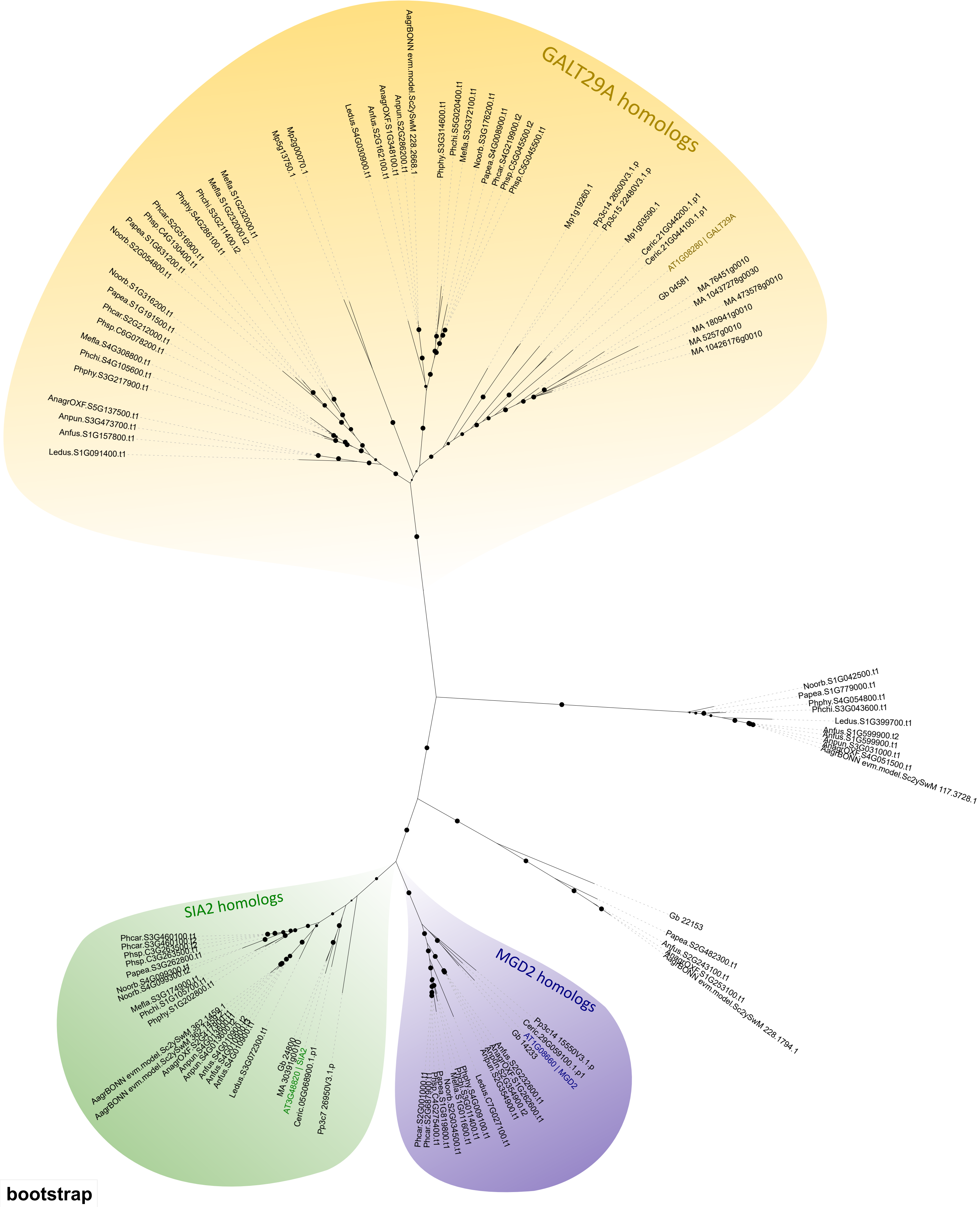

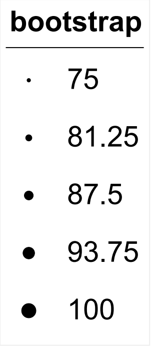

Data S4 | Phylogenetic tree for glycosyltransferase 29 (GT29) family members. Multisequence alignment was performed using MAFFT in FFT-NS-i mode and IQ-TREE to generate a maximum likelihood tree with 1000 ultrafast bootstrap replicates. The best-fit evolutionary model JTT+I+G4 was selected according to Bayesian Information Criterion.

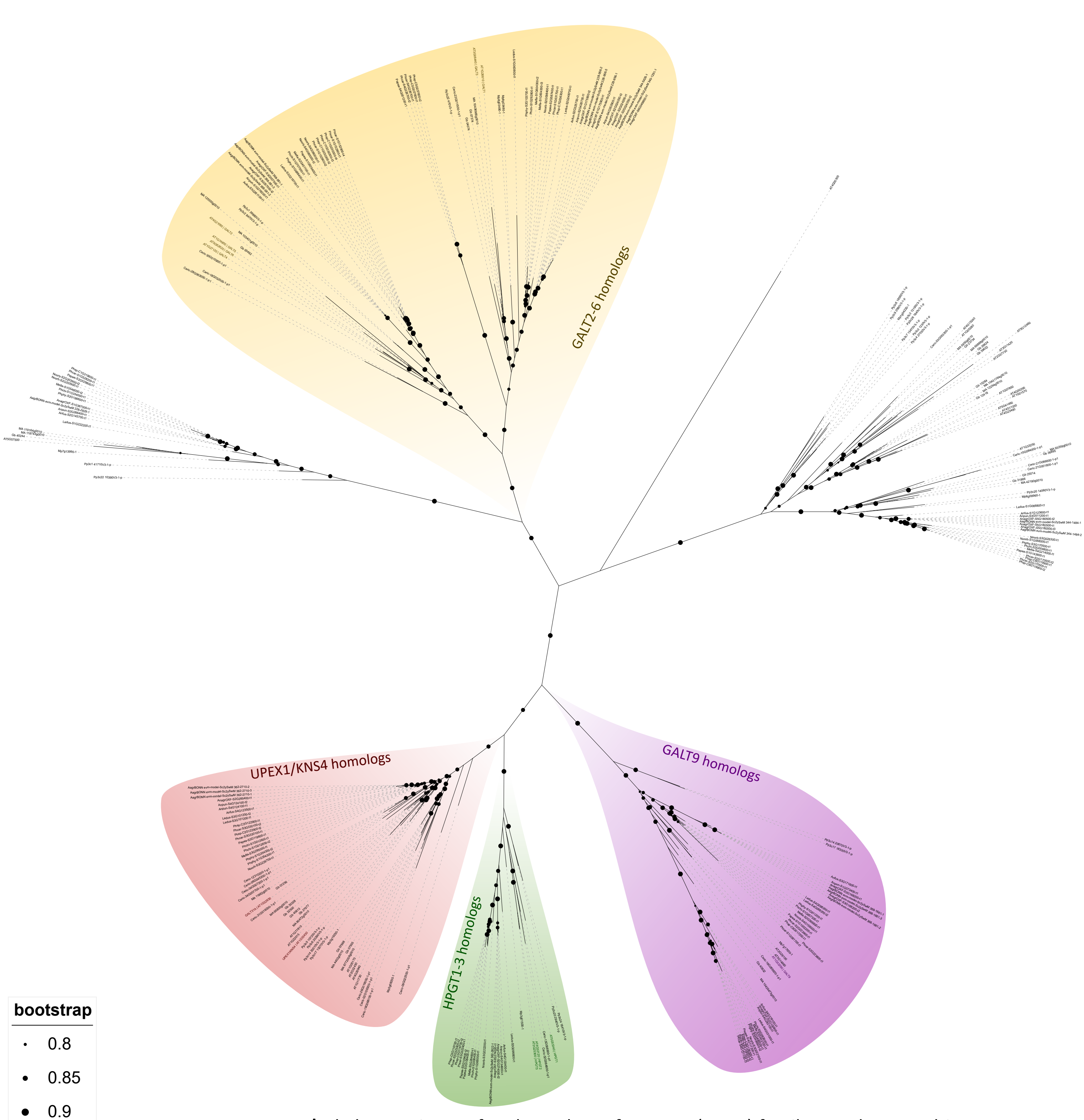

Data S5 | Phylogenetic tree for glycosyltransferase 31 (GT31) family members. Multisequence alignment was performed using MAFFT in FFT-NS-i mode and FastTree 2 to generate an approximately maximum likelihood tree. The evolutionary model JTT-CAT was chosen.

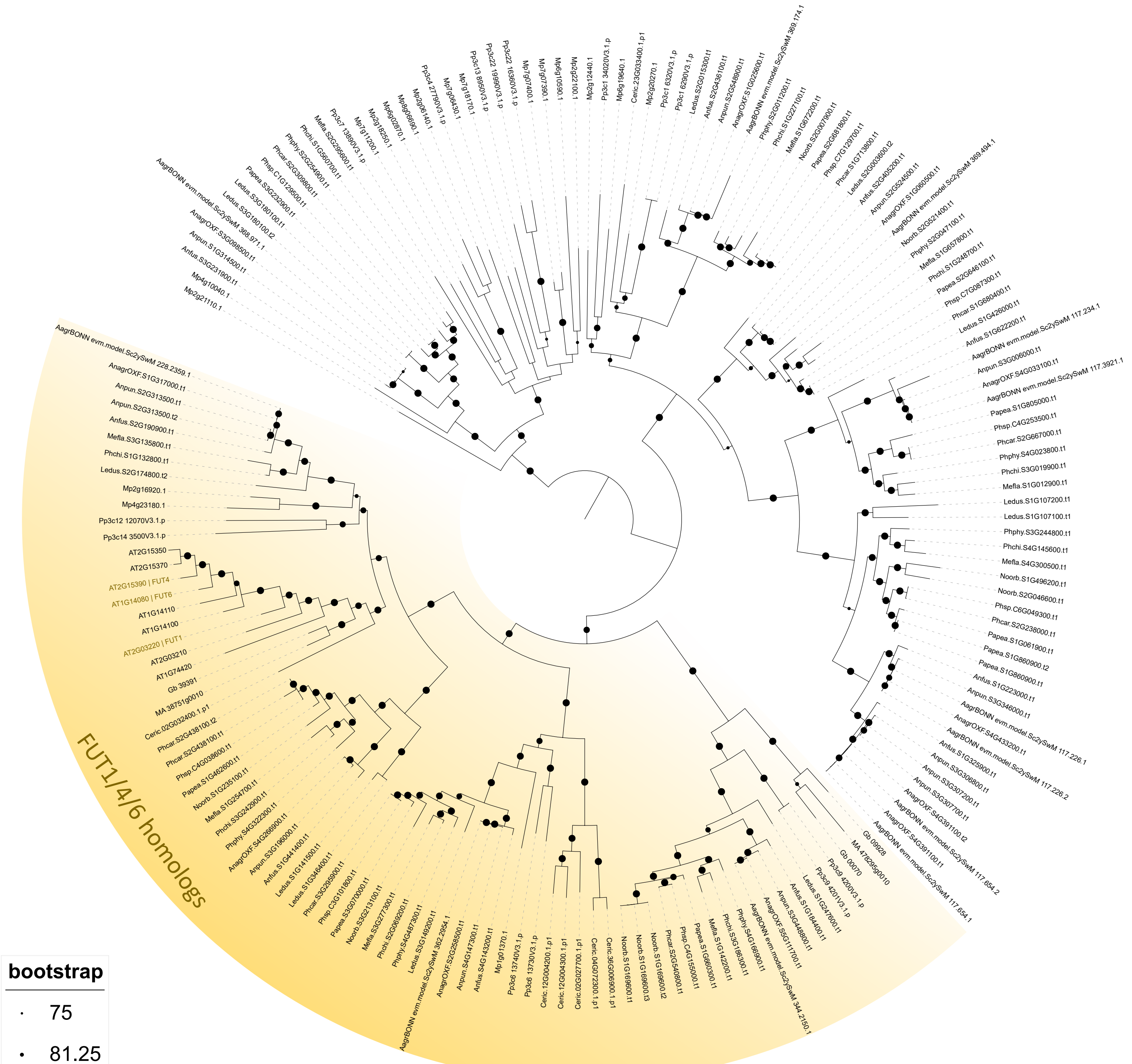

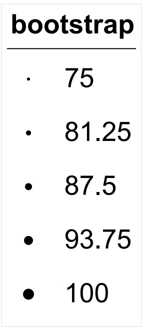

Data S6 | Phylogenetic tree for glycosyltransferase 37 (GT37) family members. Multisequence alignment was performed using MAFFT in FFT-NS-i mode and IQ-TREE to generate a maximum likelihood tree with 1000 ultrafast bootstrap replicates. The best-fit evolutionary model WAG+F+I+G4 was selected according to Bayesian Information Criterion.

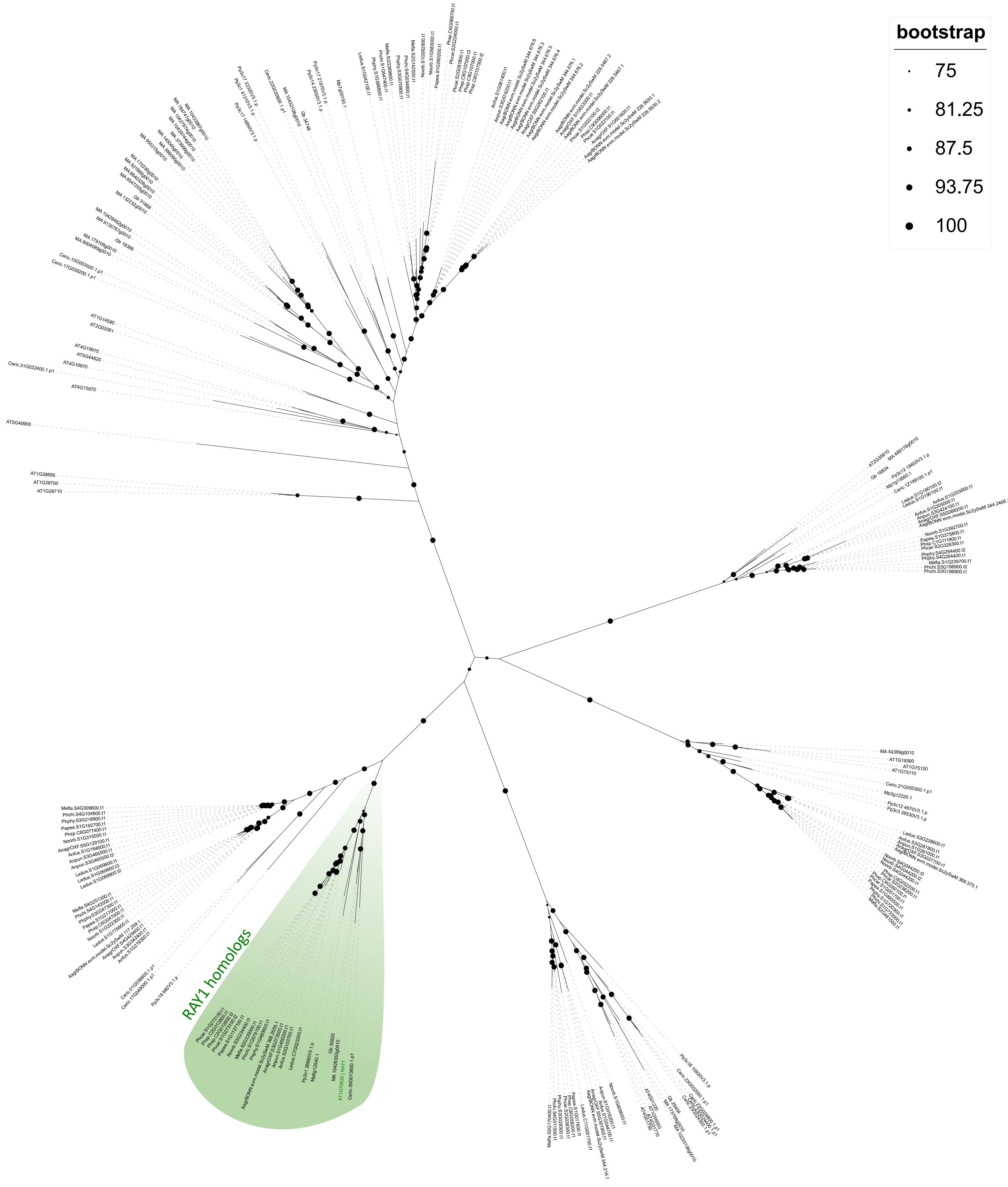

Data S7 | Phylogenetic tree for glycosyltransferase 77 (GT77) family members. Multisequence alignment was performed using MAFFT in L-INS-i mode and IQ-TREE to generate a maximum likelihood tree with 1000 ultrafast bootstrap replicates. The best-fit evolutionary model WAG+I+G4 was selected according to Bayesian Information Criterion.

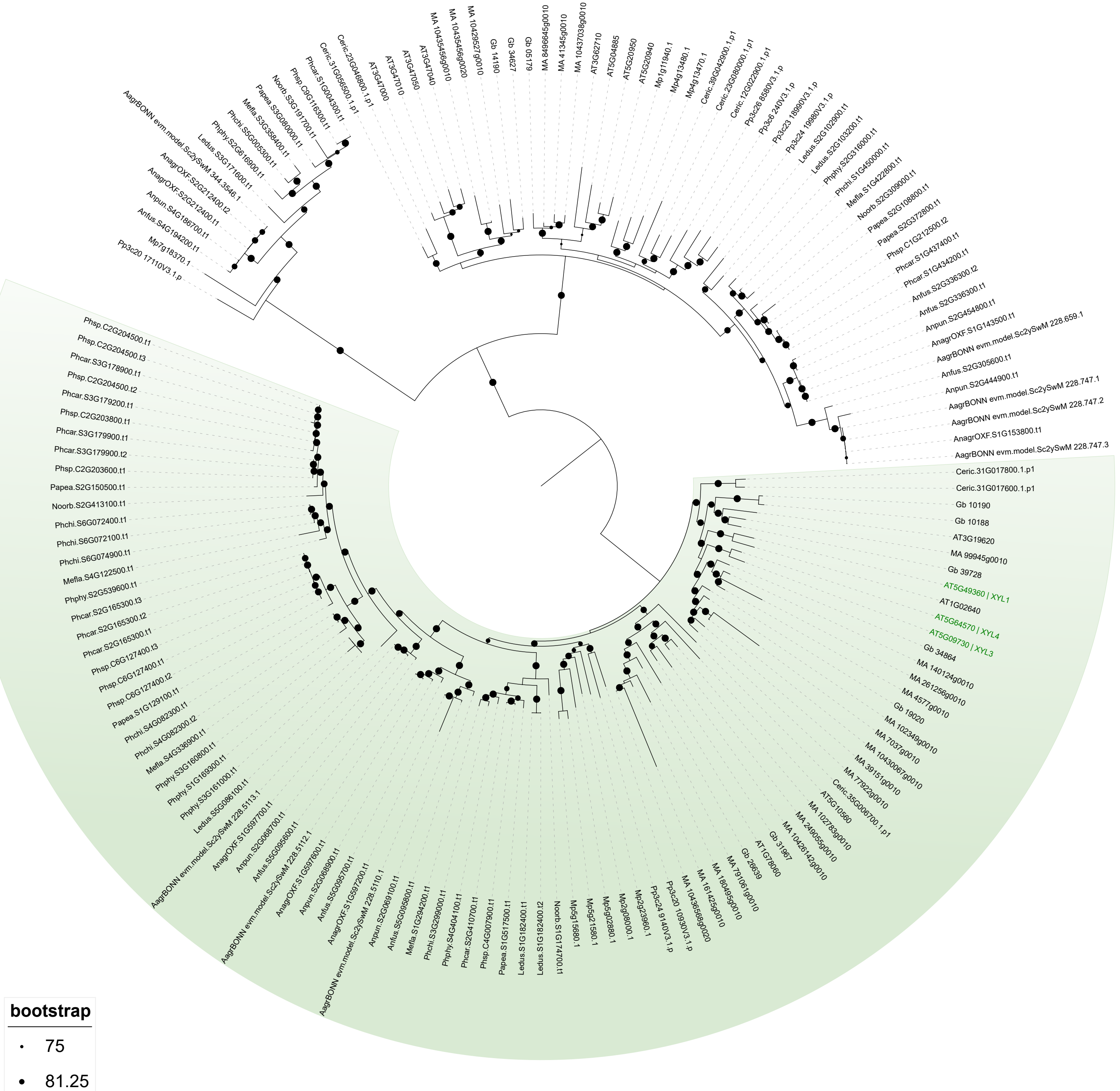

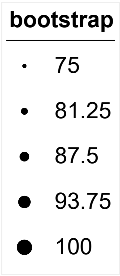

Data S8 | Phylogenetic tree for glycosylhydrolase 3 (GH3) family members. Multisequence alignment was performed using MAFFT in FFT-NS-i mode and IQ-TREE to generate a maximum likelihood tree with 1000 ultrafast bootstrap replicates. The best-fit evolutionary model WAG+I+G4 was selected according to Bayesian Information Criterion.

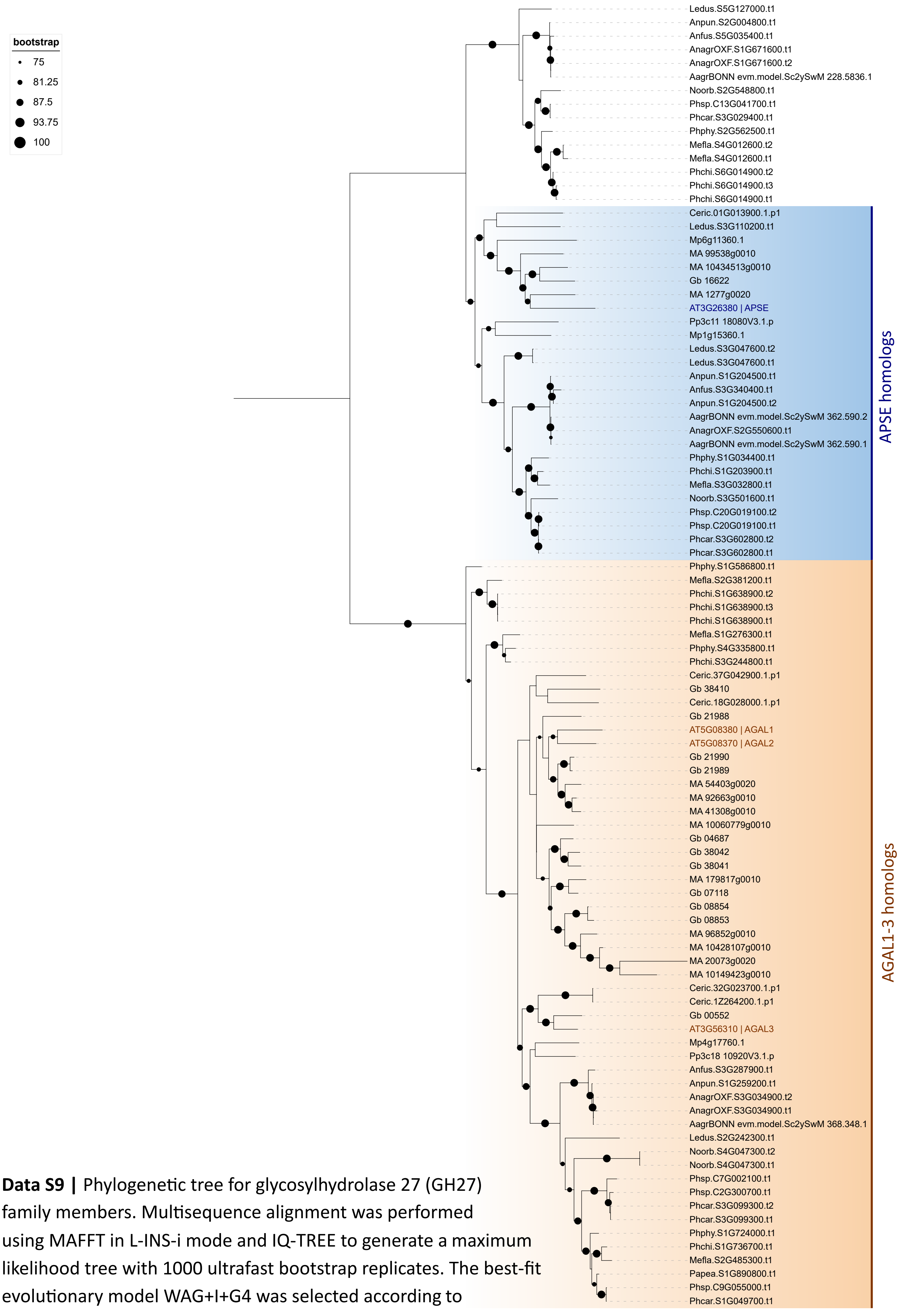

Data S9 | Phylogenetic tree for glycosylhydrolase 27 (GH27) family members. Multisequence alignment was performed using MAFFT in L-INS-i mode and IQ-TREE to generate a maximum likelihood tree with 1000 ultrafast bootstrap replicates. The best-fit evolutionary model WAG+I+G4 was selected according to Bayesian Information Criterion.

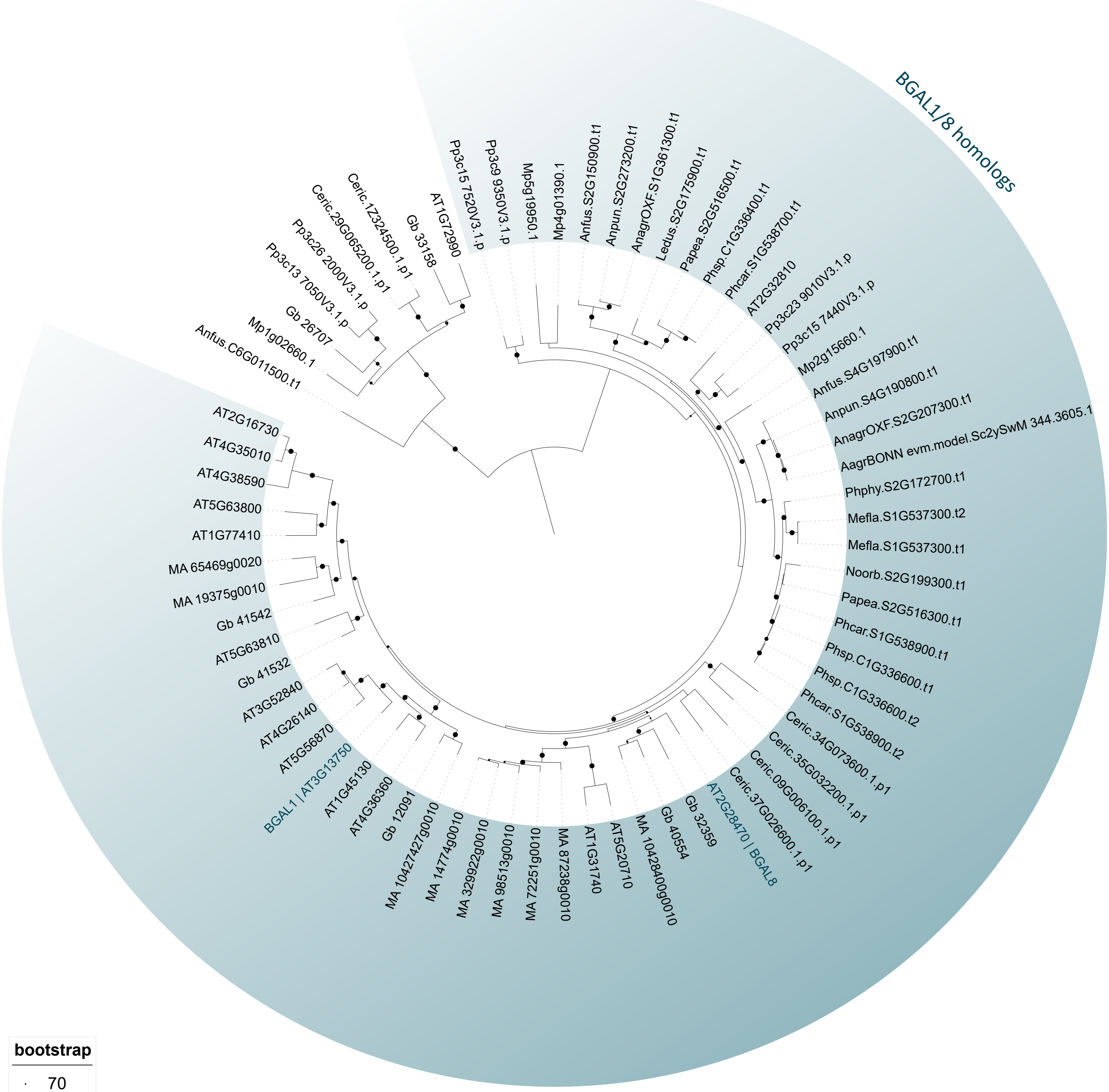

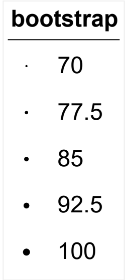

Data S 10 | Phylogenetic tree for glycosylhydrolase 35 (GH35) family members. Multisequence alignment was performed using MAFFT in L-INS-i mode and IQ-TREE to generate a maximum likelihood tree with 1000 ultrafast bootstrap replicates. The best-fit evolutionary model WAG+I+G4 was selected according to Bayesian Information Criterion.

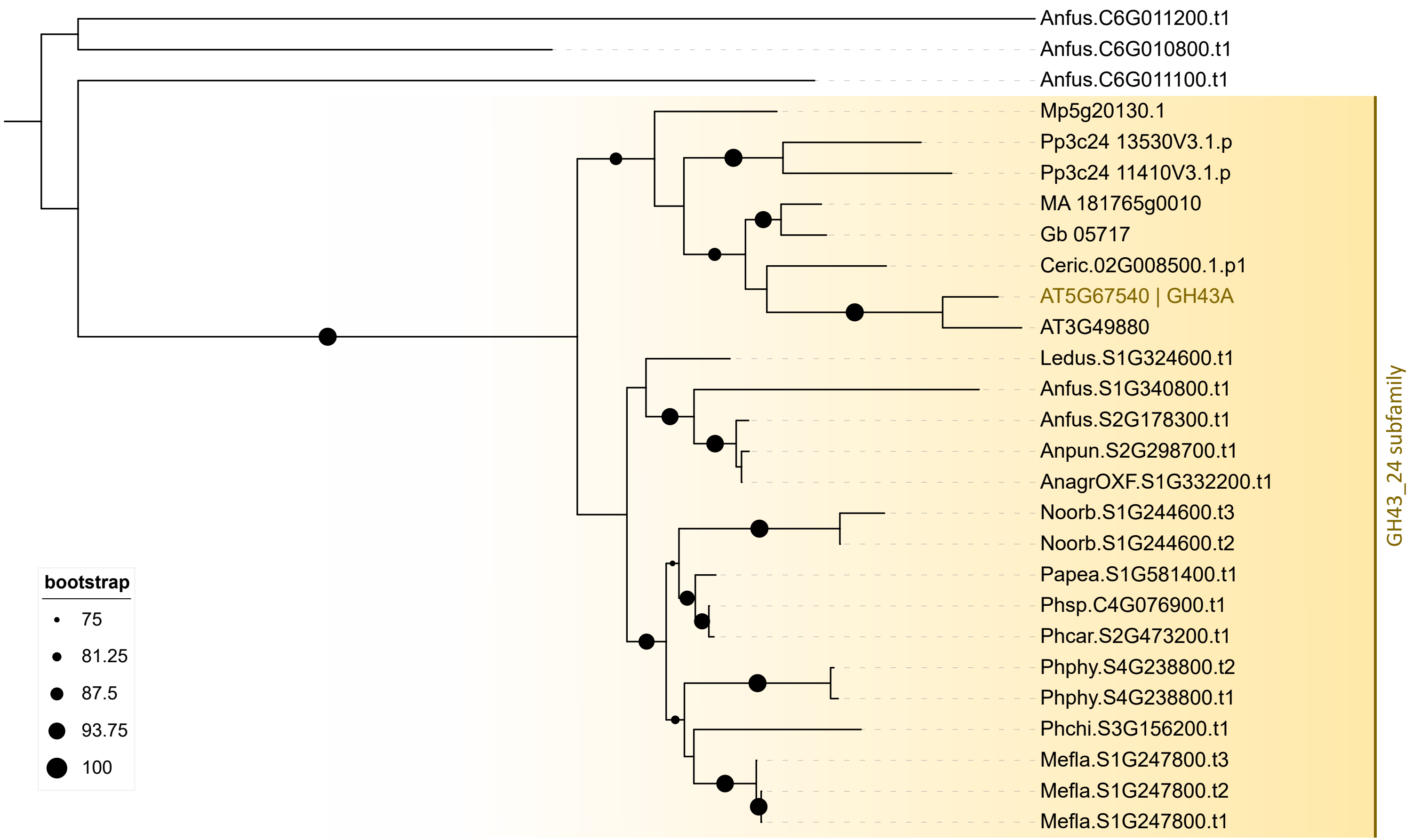

Data S 11 | Phylogenetic tree for glycosylhydrolase 43 (GH43) family members. Multisequence alignment was performed using MAFFT in L-INS-i mode and IQ-TREE to generate a maximum likelihood tree with 1000 ultrafast bootstrap replicates. The best-fit evolutionary model WAG+G4 was selected according to Bayesian Information Criterion.

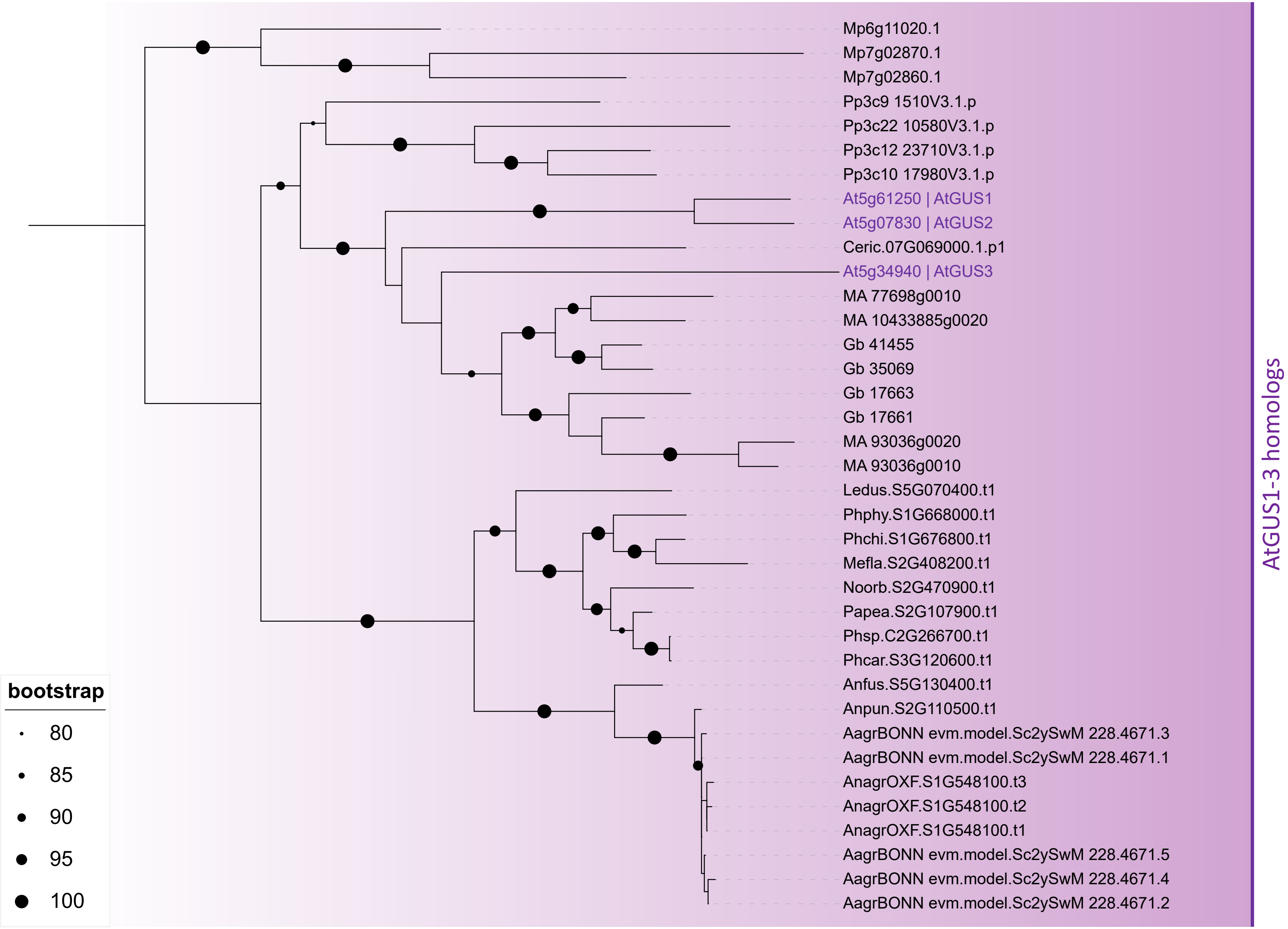

Data S 12 | Phylogenetic tree for glycosylhydrolase 79 (GH79) family members. Multisequence alignment was performed using MAFFT in L-INS-i mode and IQ-TREE to generate a maximum likelihood tree with 1000 ultrafast bootstrap replicates. The best-fit evolutionary model WAG+I+G4 was selected according to Bayesian Information Criterion.
